# Harnessing Imine Chemistry for the Debonding-on-Demand of Polyurethane Adhesives

**DOI:** 10.1021/acsami.4c19435

**Published:** 2024-12-23

**Authors:** Tankut Türel, Anna M. Cristadoro, Martin Linnenbrink, Željko Tomović

**Affiliations:** † Polymer Performance Materials Group, Department of Chemical Engineering and Chemistry, Eindhoven University of Technology, 5600 MB Eindhoven, The Netherlands; ‡ BASF Polyurethanes GmbH, Elastogranstrasse 60, 49448 Lemfoerde, Germany

**Keywords:** debonding-on-demand, imine, polyurethane, adhesive, one-component system

## Abstract

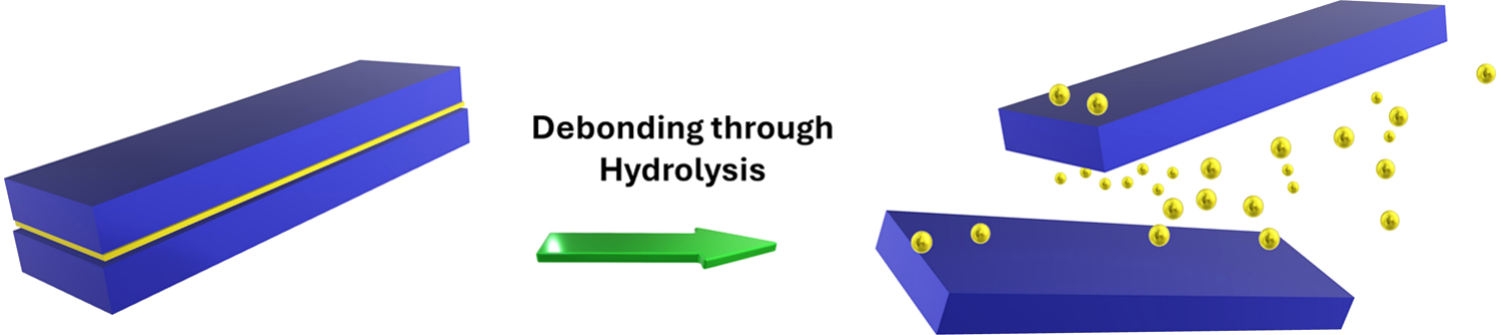

Traditional adhesives often result in irreversible bonds, hindering disassembly and recycling processes. In response to the growing demand for sustainable practices, researchers have explored alternative bonding solutions. Debonding-on-demand adhesives represent a breakthrough, enabling selective weakening or breaking of adhesive bonds when desired and facilitating efficient disassembly, repair, and recycling of bonded materials. This has profound implications across industries, including packaging, footwear, automotive, and electronics, where the recycling of bonded components plays a crucial role in resource conservation. Herein, we present the incorporation of an imine-based polyol derived from vanillin and polyetheramine into a model, one-component, polyether-based polyurethane adhesives. Such systems retained their thermal performance, exhibiting onset degradation temperatures of ∼320 °C and glass transition temperatures of ∼−50 °C, similar to the reference adhesive. Additionally, they demonstrated adhesive performance comparable to that of the reference system, with lap-shear strengths ranging from 1.7 to 2.1 MPa. In addition to that, imine polyol incorporated adhesives offered enhanced recyclability through on-demand debonding of the bonded substrates to obtain adhesive-free surfaces by acidic hydrolysis of the imine functional groups. These findings undoubtedly contribute to the advancement of sustainable material practices and promote a circular economy, emphasizing the importance of adhesive technology in addressing environmental challenges.

## 1 Introduction

A large variety of industrial products comprise diverse materials and undergo assembly and joining processes for their completion. Among the commonly used joining methods, such as welding, mechanical fastening, and adhesive bonding, adhesive bonding stands out for its ability to join dissimilar materials, distribute stress evenly, and maintain the integrity of the substrates being joined, in addition to offering simplicity and cost-effectiveness.^1,2^ However, traditional adhesives typically yield irreversible bonds, making disassembly and recycling difficult or impossible without damaging the bonded materials.^3^

The growing demand for sustainable practices and resource conservation has driven researchers to seek alternative bonding solutions that enable the separation of bonded components without compromising their integrity, aligning with the imperative for environmentally conscious practices in industry. The debonding-on-demand adhesives represents a significant advancement in adhesive technology, holding profound implications across diverse industries.^4^ These pioneering adhesives present a transformative solution to the challenges inherent in conventional permanent bonding methods, as they enable reversible bonding and debonding of materials under controlled conditions.^5−9^ By selectively weakening or breaking adhesive bonds as needed, debonding-on-demand adhesives address the limitations of traditional adhesives. This breakthrough enables efficient disassembly, repair, and recycling of bonded materials, marking a significant leap forward in adhesive technology.^10,11^ The demand for debonding-on-demand solutions spans various fields. For instance, high-performance sport shoes utilize thermosetting polyurethanes as a base, bonded to materials such as ethylene-vinyl acetate, polyester textiles, or synthetic leather and their separation at the end of service-life is required for efficient recycling.^12^ Similar requirements arise in diverse technical domains to facilitate the separation and recycling of different materials such as car seats, instrument panels, dashboards, and food packaging.^12^ Recycling bonded substrates is essential for sustainable resource management across industries, diverting materials from landfills, conserving resources, and contributing to the circular economy by reintroducing materials into production cycles, thus minimizing environmental impact and maximizing economic efficiency.^13−16^

In response to these demands, researchers have been exploring innovative approaches to developing adhesive formulations that offer debonding-on-demand capabilities. Thermal debonding methods have been widely employed, with Diels–Alder and oxime chemistry being utilized in many adhesive formulations.^17−27^ es relies on the breaking of adhesives at elevated temperatures into low-molecular-weight fragments with reduced bonding strength.^24,25^ In addition to the incorporation of specific chemistries into the adhesive composition, there are also thermally active additives capable of facilitating debonding. In this context, thermally expandable microcapsules have been also widely exploited in various formulations such as epoxy and polyurethane adhesives.^28−31^ These particles are generally microcapsules comprising a thermoplastic shell filled with liquid hydrocarbons.^32^ Accordingly, when the temperature increases, the inner hydrocarbon vaporizes and the outer shell softens, leading to the expansion and softening of the adhesive layer.^28−31^ There are also some hybrid strategies for debonding-on-demand that involve heating in combination with another stimuli. Thoma and Schubert, for example, explored such dual chemical and physical debonding approaches by combining furan-maleimide Diels–Alder adducts with thermally expandable microspheres in poly(urethane-urea) adhesives. Upon heating to 150 °C, the shear strength of the adhesive was reduced by 71%.^33^ Greenland and co-workers have introduced composite polyurethane adhesives that employ hysteresis heating in an oscillating magnetic field for debonding-on-demand. These systems, which incorporate iron oxide particles, demonstrated rapid debonding times (as low as 30 s), offering a promising approach to further enhance the functionality of thermally responsive adhesives.^34^ Nevertheless, thermal debonding methods often require high temperatures, which could limit their applicability. For instance, certain substrates (e.g., electronic components) may not withstand such elevated temperatures.^35^

In addition to thermally responsive adhesives, alternative debonding solutions exist that do not require high temperatures. Supramolecular adhesives employing reversible H-bonding moieties, host–guest interactions, metal–ligand interactions, and electrostatic interactions have also been investigated in numerous formulations. Yet, their adhesive strength has been limited so far, and these adhesives tend to be sensitive to moisture, primarily due to the weak noncovalent interactions.^7,36−38^ Light-responsive adhesives, on the other hand, (e.g., adhesives based on *E-* to *Z*- isomerization of azobenzene moieties, photoreversible reactions, or photodegradable units etc.) while offering nonthermal debonding, may, in some cases, face limitations of extended debonding times, ranging from seconds to as long as 24 h, and requiring high radiation intensity which constrain their effectiveness.^39−41^ Moreover, their reliance on radiation restricts their applicability solely to surfaces transparent to irradiation of interest.^35^

Conversely, the concept of adhesive breakdown triggered by chemical stimuli has garnered significant interest. Fluoride ion degradable adhesives, for example, have been extensively studied in various polyurethane formulations.^42−44^ Such systems typically incorporate a silyl-protected phenol degradable unit. Upon exposure to fluoride ions, the adhesives undergo degradation into smaller molecular weight segments, leading to a weakening of the adhesive bond. However, commercialization of such a system may not be feasible due to the complex multistep synthesis involved. Therefore, there is a high demand for debondable systems that are straightforward to develop and scalable for production.

The convergence of polyurethane (PU) chemistry and debonding-on-demand technology represents a significant advancement in adhesive science. Polyurethanes stand as one of the most versatile and widely employed conventional polymer families and are renowned for their exceptional adhesive properties and multifaceted applications across numerous industries. From construction to automotive, aerospace to healthcare, polyurethanes have long been recognized for their ability to provide strong and durable bonds in various bonding applications.^45−51^ Traditionally, once polyurethane adhesives are applied and cured, they form robust bonds that are difficult to reverse without causing damage to the bonded surfaces. While this permanence is often desired for structural integrity and long-term performance, there are scenarios where the ability to debond components without compromising their integrity is paramount.

**Figure 1:**
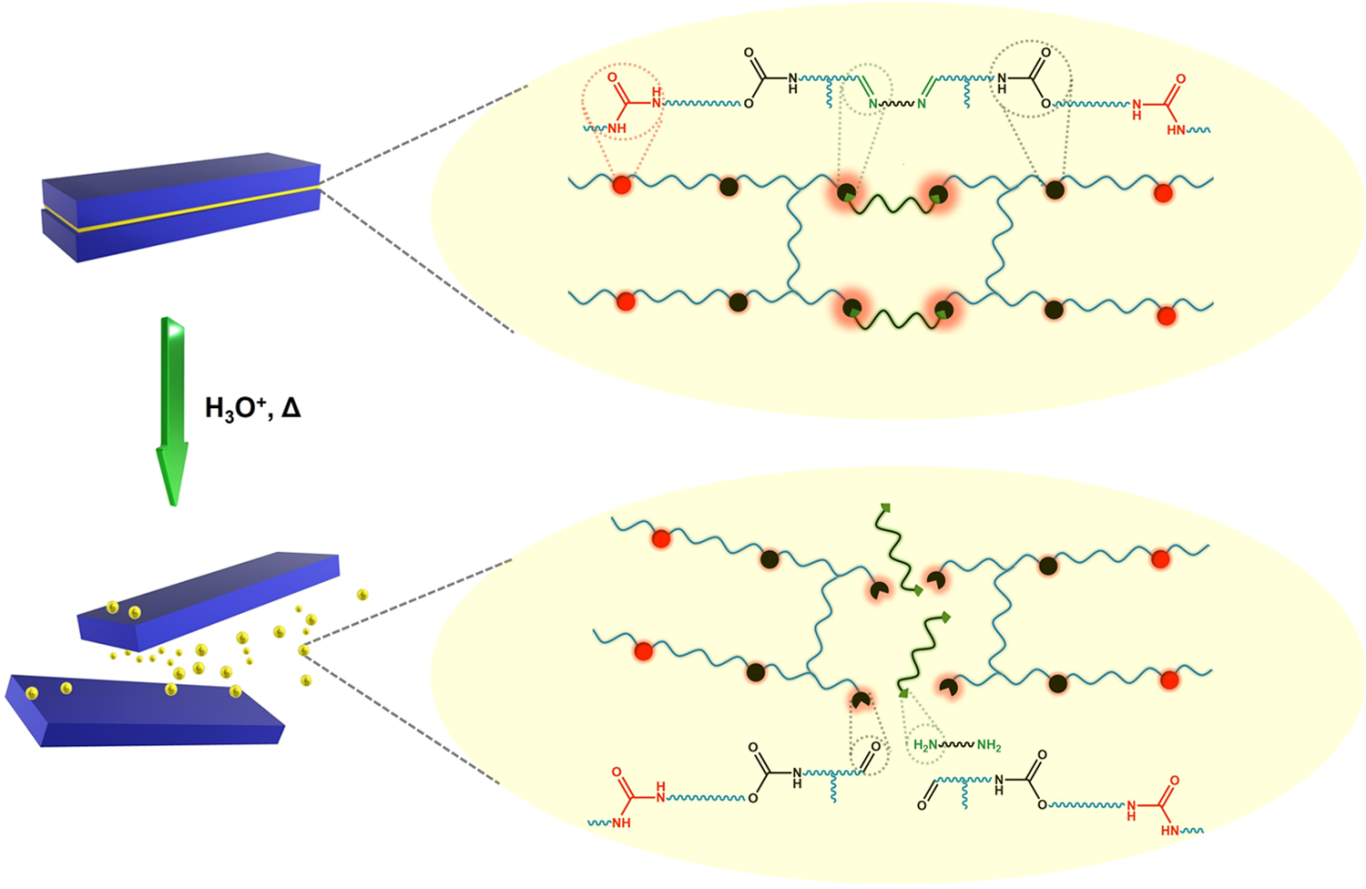
Proposed depolymerization and debonding-on-demand of **ImP** incorporated 1K PU systems through acid catalyzed hydrolysis. Detailed curing and hydrolysis schemes can be found in Supporting Information Scheme S1 and S2.

Incorporating imine functionality into polyurethanes holds significant promise for enhancing their recyclability and contributing to a more sustainable approach to materials design and utilization.^52^ Imine bonds, known for their cleavability and dynamic covalent nature, can efficiently undergo hydrolysis reactions.^53−56^ This inherent characteristic makes them particularly appealing for applications where controlled bond cleavage and reformation are crucial, such as in recycling processes. However, challenges arise when residual adhesive remains on substrates after debonding, hindering recycling efforts. Hence, a strategically designed adhesive is essential for debonding-on-demand and obtaining adhesive-free substrates subsequent to debonding. To address this, we have developed imine-polyol (**ImP**) incorporated one-component polyurethane (1K-PU) adhesives with low cross-link density (Figure 1). This carefully designed formulation ensures that residual adhesive, after debonding through hydrolysis, exhibits solubility in common organic solvents, which will facilitate the proper recycling of the substrates. Such systems retained their thermal performance, exhibiting onset degradation temperatures of ∼320 °C and glass transition temperatures of ∼ −50 °C, similar to the reference adhesive. Additionally, they demonstrated adhesive performance comparable to the reference system, with lap-shear strengths ranging from 1.7 to 2.1 MPa. Furthermore, these systems exhibited exceptional stability in water, even at elevated temperatures. In contrast, under mildly acidic conditions, they could be readily hydrolyzed, facilitating the easy debonding of bonded substrates (Figure 1). This research demonstrates a drop-in solution to the problems associated with the traditional adhesives through utilizing imine-based polyols in adhesive formulations, providing a pathway toward adhesive systems that combine robust performance with enhanced recyclability. By harnessing the reversible nature of imine bonds, these adhesives offer the potential for efficient disassembly and recycling, contributing to the advancement of sustainable materials practices and the promotion of a circular economy.

## 2 Experimental Section

### 2.1 Materials

Vanillin (99%), ethylene carbonate, Jeffamine D2000 (*M*_n_ ∼ 2000), 1,4-Diazabicyclo[2.2.2]octane (DABCO, ≥ 99%), orthophosphoric acid (H_3_PO_4_, 99%), and citric acid (≥99.5%) were procured from Merck and utilized without further purification. Na_2_CO_3_ (anhydrous, 99.5%) was purchased from Acros Organics. Deuterated CDCl_3_ was obtained from Cambridge Isotope Laboratories. Ethyl acetate (EA), chloroform, tetrahydrofuran (THF), dimethylformamide (DMF), dimethyl sulfoxide (DMSO), acetone, and *n*-hexane were procured from Biosolve B.V. and used without further purification. 2-methyltetrahydrofuran (Me-THF) was purchased from TCl Europe. Methylene diphenyl diisocyanate (MDI, Lupranat ME), Lupranol 1005/1 (polypropylene glycol with an average molecular weight of 4000 and OH number of 28 mg KOH/g), Lupranol 1000/1 (polypropylene glycol with an average molecular weight of 2000 and OH number of 55 mg KOH/g), and Lupranol 2095 (trifunctional reactive polyether polyol containing primary hydroxyl groups with an average molecular weight of 4800 and OH number of 35 mg KOH/g) were obtained from BASF (Germany) and used as received. 2,5-Bis(5-(*tert*-butyl)benzo[*d*]oxazol-2-yl)thiophene was ordered from BLDpharm and was used as a fluorescent marker to analyze the mode of failure using a fluorescent bulb operating at 365 nm. The average amine functionality of Jeffamine D2000 was determined to be 1.2 using titrimetric ^1^H NMR analysis. The amine value of Jeffamine D2000 was amounted to 33.7 mg KOH/g.

Beechwood specimens (25 mm × 100 mm) were sourced from Rocholtt and used as received. Polyethylene terephthalate (PET) foil (thickness: 0.4 mm) was procured from Rayher Hobby GmbH. Polyester-polyol-based PU foam (density: 0.58 g/cm^3^) and PET textile (GSM: 155 g/m^2^, thickness: 0.35 mm) were generously provided by BASF Polyurethanes GmbH.

### 2.2 Methods

The ^1^H and ^13^C NMR spectra were acquired on a Bruker UltraShield spectrometer operating at 400 MHz, with CDCl_3_ serving as the solvent. The FTIR spectra of the monomers, imine polyol, isocyanate prepolymers, and cured networks were recorded on a Thermo Scientific NICOLET iS20 FTIR spectrometer as an average of 8 scans over the wavenumber range of 4000–450 cm^–1^.

To determine the isocyanate content of the prepolymer, a Metrohm 916 Ti-Touch titrator was employed following the ASTM D5155-19 standard method.

Thermogravimetric analysis (TGA) was conducted on the cast films on a TA Instruments TGA 550. Samples weighing 5–10 mg underwent heating from 100 to 800 °C under a nitrogen (N_2_) atmosphere at a rate of 10 °C/min. Differential scanning calorimetry (DSC) measurements of the cast films were carried out using TA Instruments Q2000. Samples weighing 5–15 mg were placed in an Aluminum-Hermetic pan. The experiments were conducted following a classical heat/cool/heat procedure, spanning from −80 to 150 °C under a N_2_ atmosphere. The heating rate was maintained at 10 °C/min, while the cooling rate was set to 5 °C/min. Glass transition temperatures (*T*_g_) were determined by taking the midpoint of the reversible endotherm of the second heating.

Lap-shear tests were conducted with an Instron 5500R Mechanical Tester in accordance with ISO 4587/DIN EN 1465 standard, at a strain rate of 5 mm/min, utilizing a preforce of 10 N. The adhesion tests were performed using beechwood test specimens (25 mm × 100 mm, obtained from Rocholtt, Germany). To determine the experimental error, a set of three samples was employed.

The morphology following the debonding of bonded substrates or the interface between the substrates was analyzed using scanning electron microscopy (SEM, FEI Quanta 200 3D) at an acceleration voltage of 10 kV. Prior to testing, the samples were sputtered with gold for 40 s.

### 2.3 Synthesis

#### 2.3.1 Synthesis of Hydroxyethylated Vanillin

Synthesis of HEV was adopted from the reported technique with slight modification.^57^ Vanillin (30.0 g, 197 mmol, 1.00 equiv), ethylene carbonate (17.7 g, 201 mmol, 1.02 equiv), and Na_2_CO_3_ (420 mg, 3.94 mmol, 0.02 equiv) were placed in a two-neck flask equipped with a magnetic stir bar and reflux condenser. The mixture was flushed with Argon (Ar) for 30 min and then heated to 165 °C for 3 h under Ar atmosphere. After the reaction, the mixture was dissolved in chloroform and washed twice with distilled water, followed by two additional washes with brine twice to remove ethylene carbonate and sodium carbonate. The organic layer was subsequently dried over MgSO_4_ and the solvent was evaporated. Finally, HEV was purified by flash column chromatography on silica gel employing an eluent mixture of ethyl acetate/*n*-hexane (70/30). The resulting product, HEV, was obtained as a white solid, yielding 24.5 g (63%). (mp. = 95 °C). ^1^H NMR (400 MHz, CDCl_3_) δ 9.87 (s, 1H), 7.49–7.40 (m, 2H), 7.01 (d, J = 8.0 Hz, 1H), 4.22 (dd, J = 5.2, 3.9 Hz, 2H), 4.03 (ddd, J = 6.4, 5.2, 3.9 Hz, 2H), 3.93 (s, 3H), 2.30 (t, J = 6.4 Hz, 1H). ^13^C NMR (100 MHz, CDCl_3_): δ 190.9, 153.7, 150.0, 130.6, 126.7, 112.3, 109.4, 70.7, 61.1, 55.7.

#### 2.3.2 Synthesis of Imine-Based Polyol (ImP)

was mixed with Jeffamine D2000 (40.0 g, containing 12.0 mmol of amine groups). The reaction was conducted at 60 °C under a vacuum of 15 mbar for 2 h by using a rotary evaporator. Subsequently, the resulting product underwent drying for 12 h under vacuum at 40 °C. The resulting polyol **ImP** was obtained as a clear liquid. ^1^H NMR (400 MHz, Chloroform-d) δ 8.29–8.07 (m, 2H), 7.41 (d, J = 1.9 Hz, 1H), 7.15 (dd, J = 8.2, 1.9 Hz, 1H), 6.92 (d, J = 8.2 Hz, 1H), 4.23–4.09 (m, 2H), 3.96 (t, J = 4.5 Hz, 2H), 3.92 (s, 3H), 3.77–3.00 (m, 74H), 1.27–0.93 (m, 84H). ^13^C NMR (100 MHz, CDCl_3_) δ: 160.0, 150.3, 150.0, 130.7, 122.8, 113.5, 109.7, 75.4, 73.5, 73.0, 71.1, 66.1, 61.2, 56.0, 46.6, 19.8, 19.1, 18.6, 18.2, 17.5, 17.3.

#### 2.3.3 Synthesis of Reference Prepolymer

The reference prepolymer **(PPR)** was prepared from Lupranol 1005/1 (20.9 g, 5.2 mmol), Lupranol 1000/1 (11.2 g, 5.6 mmol), and Lupranol 2095 (2.3 g, 0.5 mmol). These three polyols were mixed with DABCO (65 mg, 0.57 mmol) and heated to 80 °C under Ar flow. Subsequently, MDI (7.15 g, 28.6 mmol) was introduced, and the reaction was allowed to proceed for 3 h. The experimental NCO content was 3.24%.

#### 2.3.4 Synthesis of Imine-Polyol Containing Prepolymers PP1, PP2, and PP3

Imine polyol **ImP** was incorporated into the isocyanate prepolymers by replacing either Lupranol 1000/1 or Lupranol 1005/1 in the reference prepolymer, tailored to the OH value of the polyols. This process ensures the preservation of an identical NCO content compared to the reference prepolymer.

**PP1:** The prepolymer **PP1** was prepared from Lupranol 1005/1 (20.9 g, 5.2 mmol), **ImP** (12.2 g, 5.2 mmol), and Lupranol 2095 (2.3 g, 0.5 mmol). These three polyols were mixed with DABCO (65 mg, 0.57 mmol) and heated to 80 °C under Ar flow. Subsequently, MDI (7.15 g, 28.6 mmol) was introduced, and the reaction was allowed to proceed for 3 h. The experimental NCO content of **PP1** was 3.14%.

**PP2:** The prepolymer **PP2** was prepared from Lupranol 1005/1 (5.5 g, 1.4 mmol), Lupranol 1000/1 (11.2 g, 5.6 mmol), **ImP** (12.2 g, 5.2 mmol), and Lupranol 2095 (2.3 g, 0.5 mmol). These four polyols were mixed with DABCO (65 mg, 0.57 mmol) and heated to 80 °C under an Ar flow. Subsequently, MDI (7.15 g, 28.6 mmol) was introduced, and the reaction was allowed to proceed for 3 h. The experimental NCO content of **PP2** was 3.00%.

**PP3:** The prepolymer **PP3** was prepared from Lupranol 1005/1 (20.9 g, 5.2 mmol), Lupranol 1000/1 (5.65 g. 2.8 mmol), **ImP** (6.1 g, 2.6 mmol), and Lupranol 2095 (2.3 g, 0.5 mmol). These four polyols were mixed with DABCO (65 mg, 0.57 mmol) and heated to 80 °C under Ar flow. Subsequently, MDI (7.15 g, 28.6 mmol) was introduced, and the reaction was allowed to proceed for 3 h. The experimental NCO content of **PP3** was 3.26%.

### 2.4 Film Preparation

Four grams portion of isocyanate prepolymer was poured into an oven-dried PTFE mold with a diameter of 6 cm and evacuated for 3 min to remove the bubbles. Subsequently, the casts were cured for 1 week at a temperature of 23 °C and a relative humidity (RH) of 60%, resulting in a film with a thickness of approximately 1.5 mm. The system’s reactivity was assessed in terms of tack-free time, which denotes the duration required for the sample to develop a dry surface. The experimental error in the tack-free time determination is about 15%.

### 2.5 Adhesion Specimen Preparation

Approximately, 100 mg of isocyanate prepolymer was evenly spread onto a substrate of interest measuring 10 mm × 10 mm, which could be either PU foam or PET foil. Next, a PET textile measuring 10 mm × 10 mm was affixed onto the adhesive-coated substrate. A weight of 700 g was applied to the adhesive assembly for 1 day, creating a corresponding pressure. Following this, the applied adhesive was allowed to cure for 1 week at a temperature of 23 °C and a RH of 60%.

For lap-shear measurements, the beechwood test specimens were bonded together with a specimen overlap of 25 mm × 25 mm, ensuring that the thickness of the adhesive layer remained ∼100 μm by employing a spacer.^58^ A weight of 1 kg was applied to the adhesive assembly for 1 day, creating a corresponding pressure. Subsequently, the adhesive between the bonded wood specimens were cured using the same procedure.

To examine the interface between bonded substrates and the mode of failure upon debonding, 1 wt % of 2,5-Bis(5-(*tert*-butyl)benzo[*d*]oxazol-2-yl)thiophene (a fluorescent marker) was dispersed in the prepolymer, as it remains unreactive toward isocyanate groups.

## 3 Results and Discussion

### 3.1 Synthesis and Characterization of Imine Polyol, ImP

**Figure 2:**
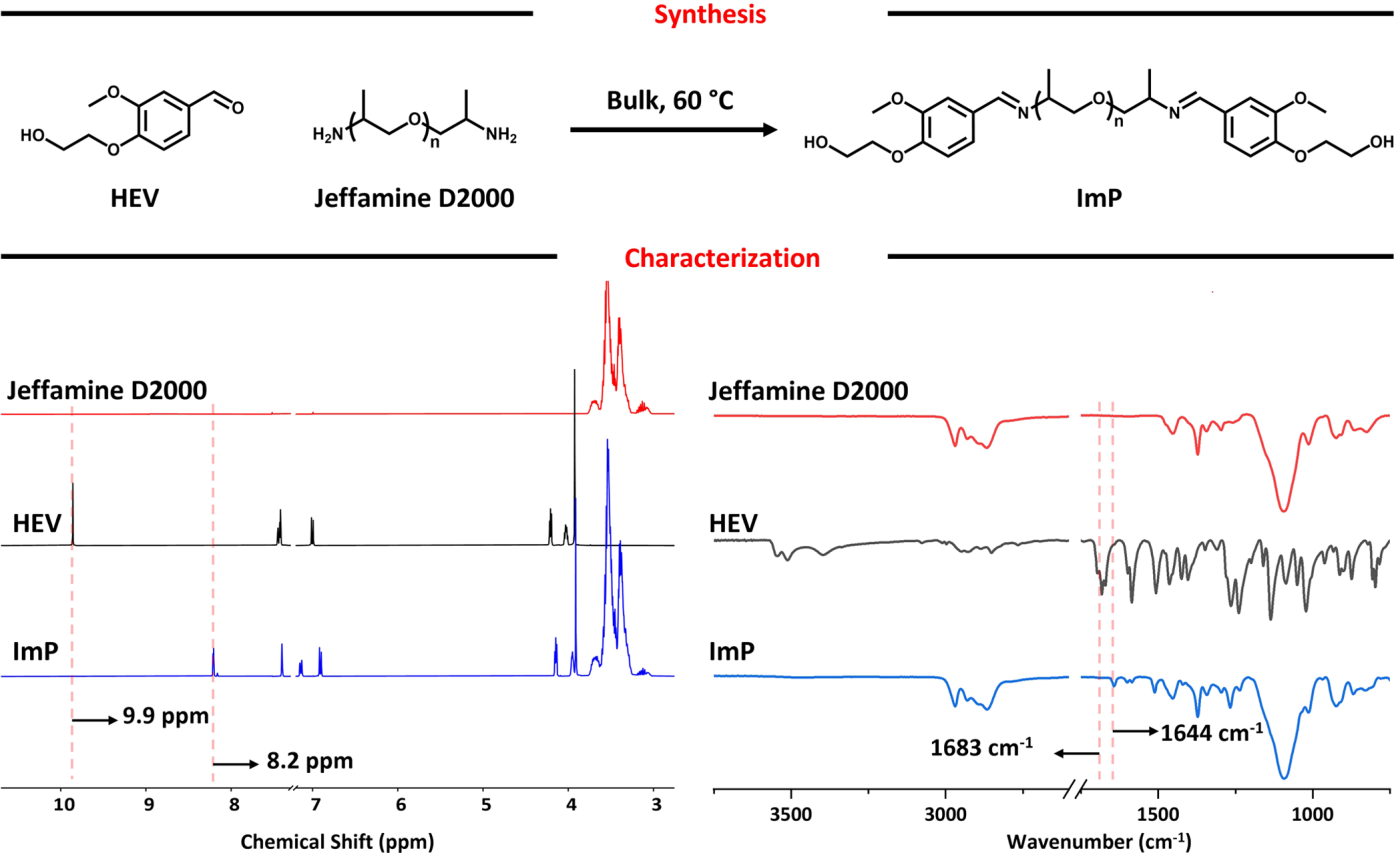
Synthetic pathway toward imine polyol, **ImP**, and its characterization via ^1^H NMR in CDCl_3_ (left) and FTIR (right).

The imine polyol **ImP** was obtained as a liquid by the straightforward, solvent-free condensation of **HEV** with Jeffamine D2000 without further purification (Figure 2). The structures of **HEV** and **ImP** were confirmed by ^1^H and ^13^C NMR, which were supported by FTIR (Figure 2, Figure S1). Imine formation was confirmed by the appearance of imine proton and carbon signals at ∼8.2 and ∼159.8 ppm, respectively, and the disappearance of the aldehydic proton and carbon resonances at 9.87 and 190.9 ppm, respectively. This was further supported by FTIR. Initially, carbonyl stretching characteristic of aldehydes was evident for **HEV** at 1683 cm^–1^. Upon imine formation, a weaker C=N stretching vibration was observed at 1644 cm^–1^.

### 3.2 Synthesis and Structural Characterization of Isocyanate Prepolymers

To validate the efficacy of imine chemistry in PU systems, reference **(PPR**) and several polyether-PU formulations incorporating **ImP** (**PP1**-**PP3**) were developed (Table 1). The **PP1**-**PP3** formulations were derived by modifying the **PPR** formulation. Accordingly, either Lupranol 1000/1 or Lupranol 1005/1 was substituted with **ImP** to obtain **PP1** or **PP2**, respectively. Moreover, we developed another prepolymer **PP3** where only half of Lupranol 1000/1 in the formulation was replaced with **ImP**, to assess its effects on debonding ability and adhesion performance.

**Table 1:** Formulations of the Prepolymers, the Experimental NCO Content, and Tack-Free Times

	**PPR**	**PP1**	**PP2**	**PP3**
Lupranol 1005/1 (g)	20.9	20.9	5.5	20.9
Lupranol 1000/1 (g)	11.2		11.2	5.65
Lupranol 2095 (g)	2.3	2.3	2.3	2.3
**ImP**(g)		12.2	12.2	6.1
MDI (g)	7.15	7.15	7.15	7.15
NCO Content (%)	3.24	3.12	3.00	3.26
tack-free time (min)	110	120	75	115

**Figure 3:**
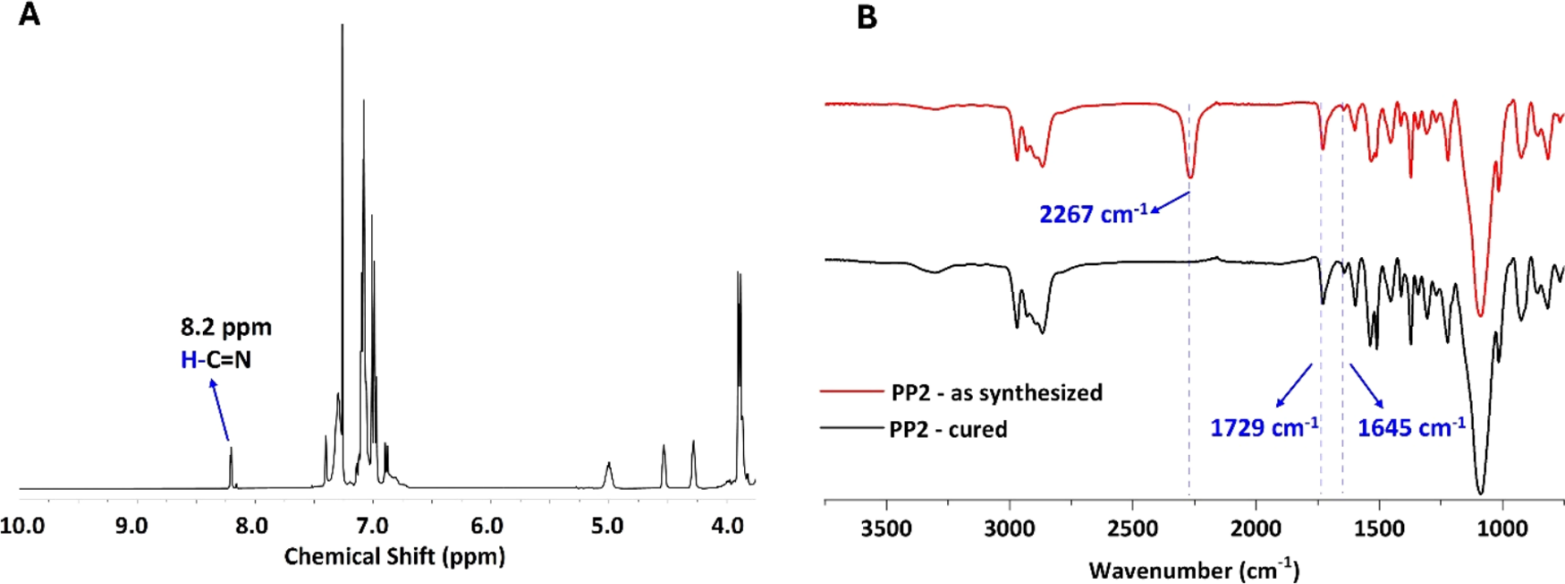
Characterization of the **PP2** before and after curing: ^1^H NMR of **PP2** prepolymer in CDCl_3_ (A), FTIR of **PP2** prepolymer, and cured network (B). The characterization of the other prepolymers can be seen in Figures S2–S5.

The isocyanate prepolymers were synthesized from a mixture of diol and triol poly(propylene oxide) polyol, along with a molar excess of 4,4′-MDI (Table 1). These prepolymers were prepared in bulk and subjected to thorough characterization via ^1^H NMR and FTIR analyses. (Figure 3, Figures S2–S5). The spectra showed the isocyanate stretching vibration (N=C=O) at 2267 cm^–1^ and a urethane carbonyl (C=O) peak at about 1729 cm^–1^.^59^ There were no detectable aldehydic proton and carbonyl stretching peaks at 9.87 ppm and ∼1680 cm^–1^ in ^1^H NMR and FT-IR, respectively. Instead, the presence of imine proton and carbonyl stretching peak at 8.2 ppm and ∼1645 cm^–1^ in ^1^H NMR and FT-IR, respectively, confirms the stability of the prepolymer and imine groups during the prepolymerization reaction (Figure 3, Figures S2–S5). Table 1 shows the formulations and tack-free times of the prepolymers. The tack-free times of **PPR**, **PP1**, and **PP3** closely align, ranging between 110 and 120 min, owing to their highly similar formulations. However, the tack-free time of **PP2** exhibited a notably shorter duration of 75 min. This disparity can be attributed to the reduced quantity of high molecular-weight polyol (Lupranol 1005/1) present in its formulation.

### 3.3 Synthesis and Characterization of Cured Polyurethane Networks

The prepolymers were cast on Teflon molds and subjected to curing at room temperature under 60% relative humidity for a week, resulting in networks that were insoluble in common solvents such as chloroform, EA, acetone, THF, DMF, and DMSO. Subsequently, the cured networks were characterized by FTIR (Figure 3, Figures S2–S6). In contrast to the spectra of the as-synthesized prepolymers, the cured networks exhibited an absence of isocyanate stretching vibration at approximately 2267 cm^–1^, confirming the complete curing of the prepolymers. The urea carbonyl stretching peak coincided with the C=N imine stretching peak at 1645 cm^–1^. Most notably, the imine functionalities remained stable throughout the curing process, as evidenced by the absence of any aldehyde stretching peak (C=O) at 1680 cm^–1^, which would indicate the hydrolysis of the imine bonds.

The fully cured castings **PPR** and **PP1–3** were submerged in THF for 1 week to assess the swelling ratios and gel contents (Table S1). Upon solvent penetration, the networks swelled to ∼1300% of their initial weight at room temperature. After drying, the gel fractions were found to be ∼85%. The relatively high swelling ratios and low gel contents can be attributed to their lightly cross-linked architectures, which was measured to be ∼20 mol/m^3^ for **PPR** and **PP1–3**.^51^

**Figure 4:**
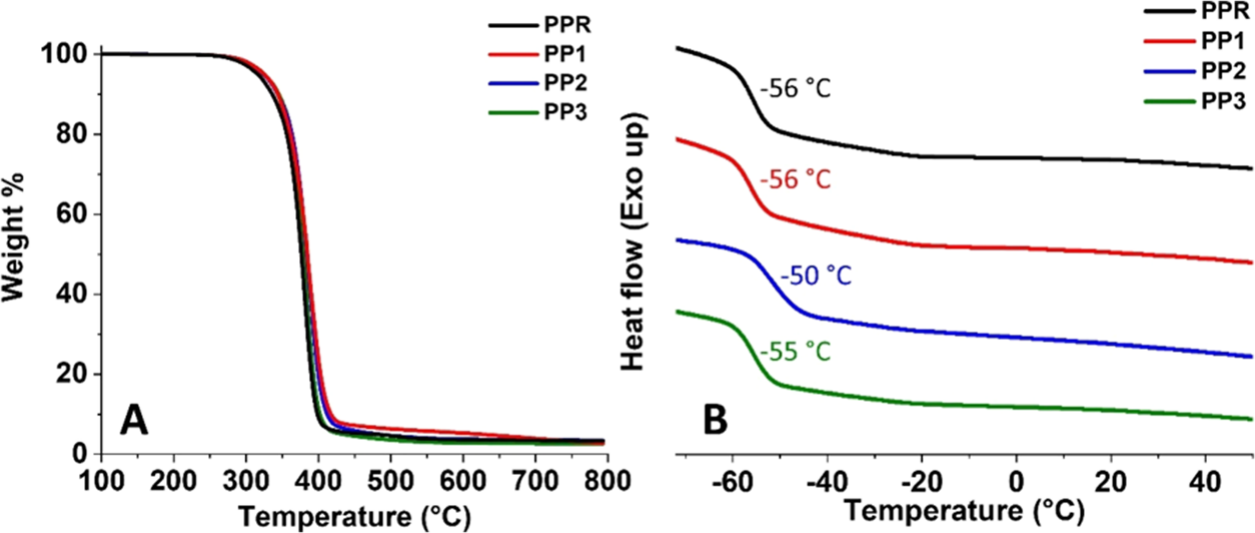
Thermal characterization of the PU networks: Thermogravimetric analysis (A) and differential scanning calorimeter (B).

Thermal stability of the networks was conducted by using TGA (Figure 4A). Specifically, **PPR** exhibited *T*_d5%_ and *T*_d30%_ values at 316 and 365 °C, respectively. Similarly, **PP1–3** demonstrated *T*_d5%_ and *T*_d30%_ within the ranges of 323–325 °C and 370–372 °C, respectively. A very minor increase observed in the *T*_d5%_ and *T*_d30%_ values could be attributed to the slightly higher aromatic content present in **PP1–3** compared with the reference (Figure 4A). Additionally, the glass transition temperatures (*T*_g_) of the materials were determined by using DSC (Figure 4B). It was found that the *T*_g_ values of **PPR** and **PP1**-**3** were identical at −56 °C. Furthermore, a slightly higher *T*_g_ of −50 °C was observed in **PP2**. This minor variance could once again be attributed to the lower quantity of high molecular-weight Lupranol 1005/1 utilized in its formulation.

### 3.4 Utilization of Isocyanate Prepolymers as Adhesives

Moisture-cured adhesives are widely used to bond porous materials, where the porosity allows the humidity to reach the bonding site and cure the prepolymer. These adhesives are versatile, bonding porous materials to themselves (e.g., wood-to-wood) or nonporous substrates (e.g., wood-to-metal or wood-to-plastic). In this study, the prepolymers were formulated to bond various substrates, including wood-to-wood, PU foam-to-PET textile, and PET foil-to-PET textile. Wood-to-wood specimens were specifically prepared for lap-shear strength testing, while the other substrates were used to evaluate the debonding ability of the adhesive networks under defined conditions (*vide infra*).

**Figure 5:**
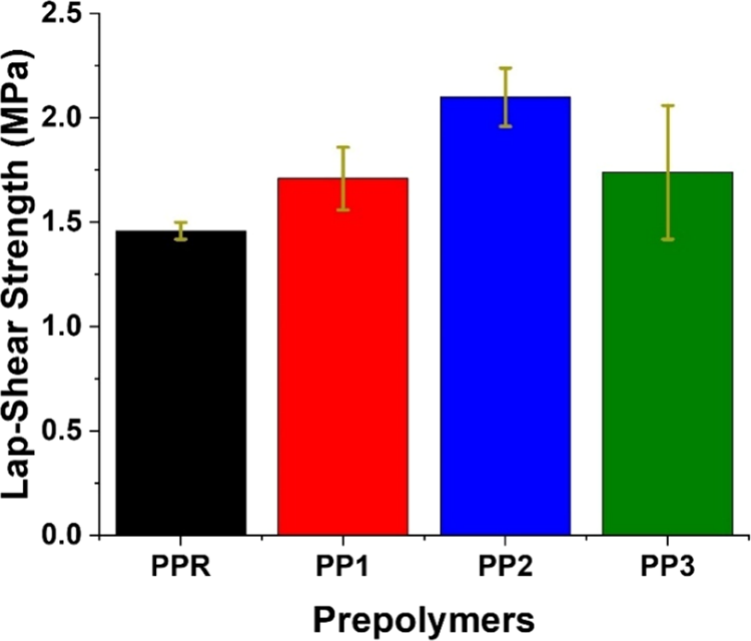
Lap-shear strength (wood–wood) of the cured prepolymers.

The lap-shear analysis of the cured adhesives revealed that all networks exhibited adhesion performance within a similar range of 1.5–2.1 MPa (Figure 5 and Figures S7–S10). There was a slight enhancement in the lap-shear strength of **PP1**, **PP2**, and **PP3**, with values of 1.71, 2.10, and 1.74 MPa, respectively, compared to that of **PPR**, which measured at 1.46 MPa. Notably, **PP2** demonstrated the highest lap-shear strength, which could be correlated with its slightly higher hard segment content (∼19%) and therefore stronger H-bonding in comparison to the other networks (hard segment content is ∼17%). Additionally, the higher proportion of lower molecular weight polyols in **PP2** may also account for the increased lap-shear strength in this formulation.

When evaluating adhesive performance, it is crucial to examine the failure mode upon the application of force. Cohesive failure occurs when the adhesive leaves residue on both surfaces post failure, while adhesive failure leaves the surfaces clean.^51^ It is imperative for the application that the adhesive exhibit cohesive failure. All systems examined in this study had a high affinity toward wood substrates and therefore displayed cohesive failure, indicating that adhesive forces surpassed the cohesive strength of the polymer.^51^

### 3.5 Depolymerization and Debonding-on-Demand Studies

The depolymerization of the films was investigated under various conditions, initially through a hydrolysis pathway using water. Despite vigorous stirring at 80 °C for 6 h, no detectable changes were observed in the structure or appearance of the films in water (Tables S2–S6, Figures S11–S15). Upon evaporation of the water, analysis of the **PP1** supernatant and residual network revealed no traces of Jeffamine D2000 or any characteristic carbonyl stretching indicative of aldehydes (Figures S12 and S13). This indicates that these networks remained highly stable to water, even at elevated temperatures.

Furthermore, we explored several acids for depolymerization and debonding studies. While HCl has been commonly used in the literature for the depolymerization of imine-based systems, its high corrosivity toward metals, skin, and respiratory tissues renders it less suitable for industrial applications. As an alternative, orthophosphoric acid is often preferred due to its lower corrosivity and toxicity. Additionally, mild organic acids (i.e., citric acid) offer a viable option. Naturally found in citrus fruits, citric acid is environmentally friendly and safe to handle. Consequently, hydrolysis studies on films and adhesive layers were conducted using either 1 M H_3_PO_4_ or 1 M citric acid at 80 °C, recognizing their suitability for industrial applications and advantages over more corrosive and toxic alternatives as HCl. The imine-based films exhibited a yellowish color upon exposure to these acids (Tables S2–S6) possibly attributed to the hydrolysis of the imines (Figures S12–S15). For instance, after 6 h, the residual network of **PP1** was notably weaker, with ^1^H NMR and FTIR analysis indicating an aldehydic proton at 9.9 ppm and carbonyl stretching vibration shoulder characteristic of aldehydes (Figure S12) and extraction of the acidic aqueous supernatant with ethyl acetate and subsequent removal of the organic solvent yielded Jeffamine D2000 (Figure S13). Additionally, the reference PU system **PPR** did not show any significant change in either its visual appearance or structure in water, 1 M H_3_PO_4_ and 1 M citric acid at 80 °C (Table S2, Figure S11). These results confirmed that imines underwent acidic hydrolysis with 1 M H_3_PO_4_ or 1 M citric acid.

To thoroughly assess the applicability of the adhesives, we conducted several adhesion tests using different substrates. One example involved replicating a high-performance sports shoe sole by bonding PU foam with PET textile through the application of these prepolymers at the interface. The debonding of the bonded substrates was achieved within 1.5–7 h at 80 °C utilizing 1 M H_3_PO_4_ or 1 M citric acid, as summarized in Supporting Information, Table S7. While imine-based adhesive layers were easily removed under acidic conditions, they remained stable in water consistent with the film depolymerization studies (Figures S16–S19). While substrates bonded with **PP1** and **PP3** debonded within similar timeframes in 1 M H_3_PO_4_ (6–7 h), **PP2** debonded significantly faster, within 3 h. This difference could be attributed to the differences in the polarity among the adhesives and the concentration of imine bonds. Specifically, prepolymers **PP1** and **PP3** with a higher concentration of the high molecular-weight polyol Lupranol 1005/1 (average molecular weight of 4000 g/mol) form fewer urethane/urea linkages upon curing as compared to the prepolymer **PP2**, which contains a higher concentration of lower molecular weight polyols, Lupranol 1000/1 and **ImP** polyol (average molecular weight of 2000 g/mol). This difference likely results in more nonpolar architectures in **PP1** and **PP3**, which hinders the hydrolyzability of the imine bonds and thereby affects the debonding duration. Moreover, the concentration of imine bonds in **PP2** (0.27 mmol/g) is higher than those in **PP1** (0.24 mmol/g) and **PP3** (0.07 mmol/g), which may contribute to the differences in debonding times. On the other hand, the debonding times were notably shorter in 1 M citric acid (up to 3 h), but the overall trend of adhesive debonding followed a similar pattern. Following debonding, the PET textile and PU foam were analyzed by using FTIR and found to be structurally identical, confirming that these substrates are suitable under debonding conditions (Figure S20).

**Figure 6:**
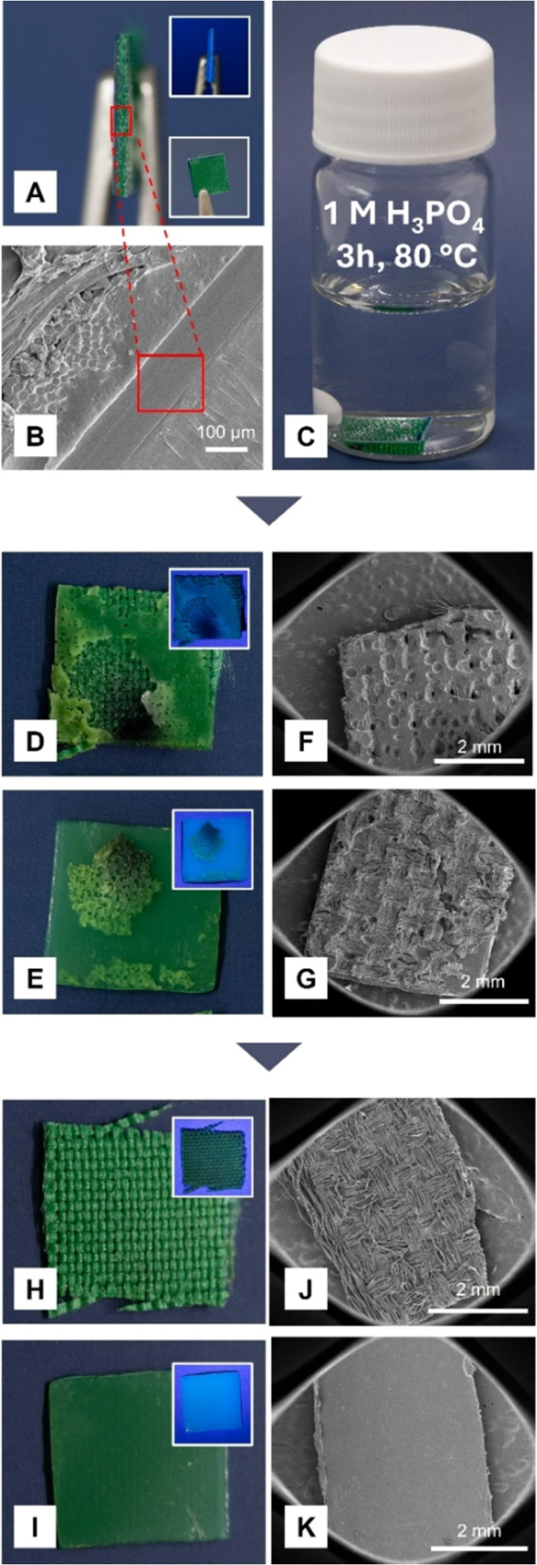
Debonding studies conducted on the **PP3**-based adhesive applied between PET foil and PET textile in 1 M H_3_PO_4_ at 80 °C. Images of the bonded substrates before acid exposure, showing the interface under normal light and UV light (upper inset) and the top surface under normal light (lower inset) (A). SEM micrograph of adhesion interface between the PET foil and PET fabric (B). Image of the bonded substrates in 1 M H_3_PO_4_ at 80 °C (*t* = 0) (C). Images of the debonded PET textile (D) and PET foil (E) substrates after 3 h of acid exposure at 80 °C under normal light and UV light (insets). SEM micrographs of the debonded PET textile (F) and PET foil (G). Images of the ethyl acetate-washed PET textile (H) and PET foil (I) under normal and UV light (insets). SEM micrographs of the ethyl acetate-washed PET textile (J) and PET foil (K).

To extend the applicability of these adhesives and understand the failure mode, we conducted another set of bonding-debonding tests using PET foil and PET textile (Figures 6 and S21 and S22). For these experiments, we employed **PP3** as the adhesive between the substrates due to its lowest imine content among all systems and our intention to theoretically examine the most challenging scenario, especially considering that **PP2** is more readily hydrolyzed (*vide supra*). Consistent with the previous results, water did not affect the adhesion; however, the substrates were successfully debonded within a couple of hours when exposed to 1 M H_3_PO_4_ and 1 M citric acid. To evaluate the adhesion under these conditions, we performed SEM analysis. Accordingly, while the interface between the substrates remained intact in water even after 24 h of exposure at 80 °C, we observed partial degradation of the adhesive in 1 M H_3_PO_4_ and 1 M citric acid at 80 °C due to the hydrolysis of the imine bonds. The mode of failure was identified as cohesive failure, supported by the residues present on both the PET foil and PET textile surfaces (Figures 6 and S22). Notably, after debonding, the residual material on the substrates could be readily dissolved and removed using common organic solvents (e.g., THF, EA, and Me-THF) within 30 min of stirring at room temperature, enabling complete recycling of the substrates. Further analysis of the recovered and washed PET foil and PET textile substrates by SEM and FTIR confirmed their surface and structural integrity following debonding, indicating that these substrates are stable under debonding conditions (Figure S23).

## 4 Conclusions

Herein, we have presented a straightforward, single-step method for synthesizing an imine-containing polyol that can be easily scaled up for commercial production. This polyol was then incorporated into several model 1K-PU adhesive systems. Characterization of thermal and adhesive performance revealed that the imine containing PU systems exhibited comparable and, in some cases, better properties compared to the reference systems lacking imine moieties. Making use of the advantage of imine hydrolysis under acidic conditions, debonding of the bonded substrates could be achieved using environmentally benign acids under mild conditions, and leftover adhesives on the substrates could be readily washed out by common organic solvents, facilitating their complete recycling. Last but not least, these systems displayed exceptional stability in water, even at elevated temperatures. While the developed PU adhesives show promising debonding performance, they require permeable substrates that are stable under mild acidic conditions and can withstand temperatures up to 80 °C. Future efforts could focus on broadening substrate compatibility and exploring debonding efficiency at lower temperatures to enhance the versatility of the method. Such adhesives, therefore, hold significant promise for facilitating efficient disassembly and recycling, thereby advancing sustainable materials practices and promoting a circular economy.

## Supporting Information Available

^1^H NMR, ^13^C NMR, and FTIR characterization of **ImP** polyol, isocyanate prepolymers, cured PU networks, and depolymerized PU networks; swelling ratio, gel content and cross-link density calculations of cured PU networks; lap-shear tests; visual appearance of the cured PU networks under different conditions, debonding studies (PDF)

## References

[ref1] Pocius A. V. Adhesion and Adhesives Technology: An Introduction, 4th ed.; Hanser: Munich, 2021.

[ref2] Banea M. D.; da Silva L. F. M. Adhesively bonded joints in composite materials: An overview. Proc. Inst. Mech. Eng., Part L 2009, 223, 1–18. 10.1243/14644207JMDA219.

[ref3] Banea M.; da Silva L. F. M.; Campilho R. An overview of the technologies for adhesive debonding on command. Ann. "Dunarea Jos" Univ. Galati, Fascicle XII 2013, 24, 11–14.

[ref4] Blelloch N. D.; Yarbrough H. J.; Mirica K. A. Stimuli-responsive temporary adhesives: enabling debonding on demand through strategic molecular design. Chem. Sci. 2021, 12, 15183–15205. 10.1039/D1SC03426J. 34976340 PMC8635214

[ref5] Liu Z.; Yan F. Switchable adhesion: On-Demand bonding and debonding. Adv. Sci. 2022, 9, 2200264 10.1002/advs.202200264. PMC9036041 35233988

[ref6] Bandl C.; Kern W.; Schlögl S. Adhesives for “debonding-on-demand”: Triggered release mechanisms and typical applications. Int. J. Adhes. Adhes. 2020, 99, 102585 10.1016/j.ijadhadh.2020.102585.

[ref7] Heinzmann C.; Weder C.; De Espinosa L. M. Supramolecular polymer adhesives: advanced materials inspired by nature. Chem. Soc. Rev. 2016, 45, 342–358. 10.1039/C5CS00477B. 26203784

[ref8] Hohl D. K.; Weder C. (De)bonding on Demand with Optically Switchable Adhesives. Adv. Opt. Mater. 2019, 7, 1900230 10.1002/adom.201900230.

[ref9] Srinivasan D. V.; Idapalapati S. Review of debonding techniques in adhesively bonded composite structures for sustainability. Sustainable Mater. Technol. 2021, 30, e00345 10.1016/j.susmat.2021.e00345.

[ref10] Banea M. D.; Da Silva L. F. M.; Campilho R. D. S. G.; Sato C. Smart Adhesive Joints: An overview of recent developments. J. Adhes. 2014, 90, 16–40. 10.1080/00218464.2013.785916.

[ref11] Mulcahy K. R.; Kilpatrick A. F. R.; Harper G.; Walton A.; Abbott A. P. Debondable adhesives and their use in recycling. Green Chem. 2022, 24, 36–61. 10.1039/D1GC03306A.

[ref12] Cristadoro A. M.; Kleeman J.; Spreen S.; Roeder J.; Poeselt E.; Gutmann P. Debondable Compact Pu Materials. WO Patent, 2023/242421 WO A1.

[ref13] Parchomenko A.; De Smet S.; Pals E.; Vanderreydt I.; Van Opstal W. The circular economy potential of reversible bonding in smartphones. Sustainable Prod. Consum. 2023, 41, 362–378. 10.1016/j.spc.2023.08.017.

[ref14] Lu Y.; Broughton J.; Winfield P. H. A review of innovations in disbonding techniques for repair and recycling of automotive vehicles. Int. J. Adhes. Adhes. 2014, 50, 119–127. 10.1016/j.ijadhadh.2014.01.021.

[ref15] Banea M. D. Debonding on demand of adhesively bonded joints: A critical review. In Progress in Adhesion and Adhesives; Mittal K. L., Ed.; Wiley, 2020; Vol. 5, pp 33–5.

[ref16] Banea M. D. Debonding of Structural Adhesive Joints. In Structural Adhesive Joints: Design, Analysis and Testing; Mittal K. L.; Panigrahi S. K., Eds.; Wiley, 2020; pp 135–158.

[ref17] Xi J.; Wang N. Synthesis of high mechanical strength and thermally recyclable and reversible polyurethane adhesive by Diels–Alder Reaction. Macromol. Chem. Phys. 2024, 225, 2400199 10.1002/macp.202400199.

[ref18] Wouters M.; Burghoorn M.; Ingenhut B. L. J.; Timmer K.; Rentrop C.; Bots T. L.; Oosterhuis G. J. E.; Fischer H. Tuneable adhesion through novel binder technologies. Prog. Org. Coat. 2011, 72, 152–158. 10.1016/j.porgcoat.2010.12.014.

[ref19] Wu M.; Liu Y.; Du P.; Wang X.; Yang B. Polyurethane hot melt adhesive based on Diels-Alder reaction. Int. J. Adhes. Adhes. 2020, 100, 102597 10.1016/j.ijadhadh.2020.102597.

[ref20] Ramimoghadam D.; Szmalko D.; Dilag J.; Ladani R. B.; Mouritz A. P.; Bateman S. Thermally reversible prototype adhesive via the furan–maleimide Diels–Alder reaction. Int. J. Adhes. Adhes. 2024, 128, 103522 10.1016/j.ijadhadh.2023.103522.

[ref21] Turkenburg D. H.; Van Bracht H.; Funke B.; Schmider M.; Janke D.; Fischer H. Polyurethane adhesives containing Diels–Alder-based thermoreversible bonds. J. Appl. Polym. Sci. 2017, 134, 44972 10.1002/app.44972.

[ref22] Quiles-Díaz S.; Seyler H.; Ellis G.; Shuttleworth P. S.; Gómez-Fatou M. A.; Salavagione H. J.; Salavagione H. J. Designing new sustainable polyurethane adhesives: influence of the nature and content of Diels–Alder adducts on their thermoreversible behavior. Polymers 2022, 14, 3402 10.3390/polym14163402. 36015659 PMC9414518

[ref23] Carbonell-Blasco M. P.; Moyano M. A.; Hernández-Fernández C.; Sierra-Molero F. J.; Pastor I. M.; Alonso D. A.; Arán-AíS F.; Orgilés-Calpena E. Polyurethane Adhesives with Chemically Debondable Properties via Diels–Alder Bonds. Polymers 2024, 16, 21 10.3390/polym16010021. PMC10780649 38201686

[ref24] Sridhar L.; Slark A. T.; Wilson J. A. Furan functionalized polyesters and polyurethanes for thermally reversible reactive hotmelt adhesives. In Furan Derivatives—Recent Advances and Applications; InTech Open: London, 2022.

[ref25] Kaiser K. M. A. Recycling of multilayer packaging using a reversible cross-linking adhesive. J. Appl. Polym. Sci. 2020, 137, e49230 10.1002/app.49230.

[ref26] Wang S.; Liu Z.; Zhang L.; Guo Y.; Song J.; Lou J.; Guan Q.; He C.; You Z. Strong, detachable, and self-healing dynamic crosslinked hot melt polyurethane adhesive. Mater. Chem. Front. 2019, 3, 1833–1839. 10.1039/C9QM00233B.

[ref27] Zhong K.; Guan Q.; Sun W.; Qin M.; Liu Z.; Zhang L.; Xu J.; Zhang F.; You Z. Hot-Melt adhesive based on dynamic Oxime–Carbamate bonds. Ind. Eng. Chem. Res. 2021, 60, 6925–6931. 10.1021/acs.iecr.1c00768.

[ref28] Kim D.; Chung I.; Kim G.-N. Dismantlement studies of dismantlable polyurethane adhesive by controlling thermal property. J. Adhes. Sci. Technol. 2012, 26, 2571–2589. 10.1080/01694243.2012.690213.

[ref29] Banea M. D.; Da Silva L. F. M.; Carbas R. J. C.; Campilho R. D. S. G. Mechanical and thermal characterization of a structural polyurethane adhesive modified with thermally expandable particles. Int. J. Adhes. Adhes. 2014, 54, 191–199. 10.1016/j.ijadhadh.2014.06.008.

[ref30] Nishiyama Y.; Uto N.; Sato C.; Sakurai H. Dismantlement behavior and strength of dismantlable adhesive including thermally expansive particles. Int. J. Adhes. Adhes. 2003, 23, 377–382. 10.1016/S0143-7496(03)00067-8.

[ref31] McCurdy R. H.; Hutchinson A. R.; Winfield P. H. The mechanical performance of adhesive joints containing active disbonding agents. Int. J. Adhes. Adhes. 2013, 46, 100–113. 10.1016/j.ijadhadh.2013.06.001.

[ref32] Morehouse D. S. J.; Tetreault R. J. Expandable thermoplastic polymer particles containing volatile fluid foaming agent and method of foaming the same. U.S. Patent US 3615972.

[ref33] Thoma J. L.; Elsener R.; Burgert I.; Schubert M. Chemical and Physical Debonding-on-Demand of Poly(urethane urea) Thermoset Adhesives to Facilitate the Recycling of Engineered Wooden Products. ACS Appl. Polym. Mater. 2024, 6, 5778–5787. 10.1021/acsapm.4c00439.

[ref34] Salimi S.; Babra T. S.; Dines G. S.; Baskerville S. W.; Hayes W.; Greenland B. W. Composite polyurethane adhesives that debond-on-demand by hysteresis heating in an oscillating magnetic field. Eur. Polym. J. 2019, 121, 109264 10.1016/j.eurpolymj.2019.109264.

[ref35] Avshalomov R.; Jarach N.; Dodiuk H. Breaking the unbreakable bond: Towards sustainable adhesives’ future. Eur. Polym. J. 2024, 209, 112920 10.1016/j.eurpolymj.2024.112920.

[ref36] Sun P.; Qin B.; Xu J.; Zhang X. High-Performance supramolecular adhesives. Macromol. Chem. Phys. 2023, 224, 2200332 10.1002/macp.202200332.

[ref37] Del Prado A.; Hohl D. K.; Balog S.; De Espinosa L. M.; Weder C. Plant Oil-Based supramolecular polymer networks and composites for Debonding-on-Demand adhesives. ACS Appl. Polym. Mater. 2019, 1, 1399–1409. 10.1021/acsapm.9b00175.

[ref38] Ferahian A.; Hohl D. K.; Weder C.; De Espinosa L. M. Bonding and Debonding on Demand with Temperature and Light Responsive Supramolecular Polymers. Macromol. Mater. Eng. 2019, 304, 1900161 10.1002/mame.201900161.

[ref39] Zhou Y.; Chen M.; Ban Q.; Zhang Z.; Shuang S.; Koynov K.; Butt H.-J.; Kong J.; Wu S. Light-Switchable polymer adhesive based on photoinduced reversible Solid-to-Liquid transitions. ACS Macro Lett. 2019, 8, 968–972. 10.1021/acsmacrolett.9b00459. 35619479

[ref40] Liu Z.; Cheng J.; Zhang J. An Efficiently Reworkable Thermosetting Adhesive Based on Photoreversible [4 + 4] Cycloaddition Reaction of Epoxy-Based Prepolymer with Four Anthracene End Groups. Macromol. Chem. Phys. 2021, 222, 2000298 10.1002/macp.202170003.

[ref41] Wang Y.-Z.; Li L.; Du F.-S.; Li Z.-C. A facile approach to catechol containing UV dismantlable adhesives. Polymer 2015, 68, 270–278. 10.1016/j.polymer.2015.05.032.

[ref42] Babra T. S.; Trivedi A.; Warriner C. N.; Bazin N.; Castiglione D. C.; Sivour C.; Hayes W.; Greenland B. W. Fluoride degradable and thermally debondable polyurethane based adhesive. Polym. Chem. 2017, 8, 7207–7216. 10.1039/C7PY01653K.

[ref43] Babra T. S.; Wood M. D.; Godleman J.; Salimi S.; Warriner C. N.; Bazin N.; Siviour C. R.; Hamley I. W.; Hayes W.; Greenland B. W. Fluoride-responsive debond on demand adhesives: Manipulating polymer crystallinity and hydrogen bonding to optimize adhesion strength at low bonding temperatures. Eur. Polym. J. 2019, 119, 260–271. 10.1016/j.eurpolymj.2019.07.038.

[ref44] Babra T. S.; Warriner C. N.; Bazin N.; Hayes W.; Greenland B. W. A fluoride degradable crosslinker for debond-on-demand polyurethane based crosslinked adhesives. Mater. Today Commun. 2021, 26, 101777 10.1016/j.mtcomm.2020.101777.

[ref45] Randall D.; Lee S. Polyurethanes Book; John Wiley & Sons, 2002. ISBN: 978-0-470-85041-1.

[ref46] Eling B.; Tomović Ž.; Schädler V. Current and Future Trends in Polyurethanes: An Industrial Perspective. Macromol. Chem. Phys. 2020, 221, 2000114 10.1002/macp.202000114.

[ref47] Engels H.-W.; Pirkl H.-G.; Albers R.; Albach R. W.; Krause J.; Hoffmann A.; Casselmann H.; Dormish J. Polyurethanes: Versatile Materials and Sustainable Problem Solvers for Today’s Challenges. Angew. Chem., Int. Ed. 2013, 52, 9422–9441. 10.1002/anie.201302766. 23893938

[ref48] Du H.; Zhao Y.; Li Q.; Wang J.; Kang M.; Wang X.; Xiang H. Synthesis and Characterization of Waterborne Polyurethane Adhesive from MDI and HDI. J. Appl. Polym. Sci. 2008, 110, 1396–1402. 10.1002/app.28805.

[ref49] Ates M.; Karadag S.; Eker A. A.; Eker B. Polyurethane foam materials and their industrial applications. Polym. Int. 2022, 71, 1157–1163. 10.1002/pi.6441.

[ref50] Gupta R. K.; Kahol P.; Gupta R. K. Introduction to Polyurethane Chemistry. In Polyurethane Chemistry: Renewable Polyols and Isocyanates, ACS Symposium Series; American Chemical Society: Washington, DC, 2021; Vol. 1380.

[ref51] Türel T.; Eling B.; Cristadoro A.; Mathieu T.; Linnenbrink M.; Tomović Ž. Novel Furfural-Derived Polyaldimines as Latent Hardeners for Polyurethane Adhesives. ACS Appl. Mater. Interfaces 2024, 16, 6414–6423. 10.1021/acsami.3c17416. 38282385 PMC10859888

[ref52] Zhang Z.; Lei D.; Zhang C.; Wang Z.; Jin Y.; Zhang W.; Liu X.; Sun J. Strong and Tough Supramolecular Covalent Adaptable Networks with Room-Temperature Closed-Loop Recyclability. Adv. Mater. 2023, 35, 2208619 10.1002/adma.202208619. 36367361

[ref53] Saito K.; Eisenreich F.; Türel T.; Tomović Ž. Closed-Loop Recycling of Poly(Imine-Carbonate) Derived from Plastic Waste and Bio-based Resources. Angew. Chem., Int. Ed. 2022, 134, e202211806 10.1002/ange.202211806. PMC9828757 36074694

[ref54] Türel T.; Tomović Ž. Chemically Recyclable and Upcyclable Epoxy Resins Derived from Vanillin. ACS Sustainable Chem. Eng. 2023, 11, 8308–8316. 10.1021/acssuschemeng.3c00761.

[ref55] Türel T.; Dağlar Ö.; Pantazidis C.; Tomović Ž. Chemically Recyclable and Reprogrammable Epoxy Thermosets Derived from Renewable Resources. RSC Sustainability 2024, 2, 3311 10.1039/D4SU00382A.

[ref56] Türel T.; Saito K.; Glišić I.; Middelhoek T.; Tomović Ž. Closing the Loop: Polyimine Thermosets from Furfural Derived Bioresources. RSC Appl. Polym. 2024, 2, 395–402. 10.1039/D3LP00268C.

[ref57] Liaw D. J.; Chen P. S. Preparation and properties of polyesters derived from 4, 4′-sulfonyl dibenzoyl chloride by solution polycondensation. J. Polym. Sci., Part A:Polym. Chem. 1996, 34, 885–891. 10.1002/(SICI)1099-0518(19960415)34:5<885::AID-POLA17>3.0.CO;2-E.

[ref58] Eling B.; Auffarth S.; Linnenbrink M.; Albuerne J.; Mu L. BASF SE. Adhesives Based on Carbodimide Chemistry. US Patent 2022/0153906 US A12022.

[ref59] Kim H.; Cha I.; Yoon Y.; Cha B. J.; Yang J.; Kim Y. D.; Song C. Facile Mechanochemical Synthesis of Malleable Biomass-Derived Network Polyurethanes and Their Shape-Memory Applications. ACS Sustainable Chem. Eng. 2021, 9, 6952–6961. 10.1021/acssuschemeng.1c00390.

