# Supplementary material for: Harnessing Imine Chemistry for the Debonding-on-Demand of Polyurethane Adhesives
Source: ACS Appl Mater Interfaces. 2024 Dec 23;17(1):2656–65. doi: 10.1021/acsami.4c19435 (PMC11783524; doi:10.1021/acsami.4c19435)
Supplement: Supplementary file 1 — am4c19435_si_001.pdf [file am4c19435_si_001.pdf]

Supporting Information

Harnessing Imine Chemistry for the Debonding-on-Demand of Polyurethane Adhesives

Tankut Türel,[a] Anna M. Cristadoro,[b] Martin Linnenbrink,[b]* and Željko Tomović[a]*

a) Polymer Performance Materials Group, Department of Chemical Engineering and Chemistry, Eindhoven University of Technology, 5600 MB Eindhoven, The Netherlands.

b) BASF Polyurethanes GmbH, Elastogranstrasse 60, Lemfoerde, 49448, Germany.

*E-mail: *martin.linnenbrink@basf.com; *z.tomovic@tue.nl

Table of Contents

1 . Characterization of imine polyol, ImP S2
2 . Synthesis and characterization of prepolymers and cured networks S3
3. Swelling ratio, gel content and crosslink densities S8
4. Lap-shear tests S10
5. Depolymerization studies S12
6. Debonding studies S19

1 . Characterization of imine polyol, ImP

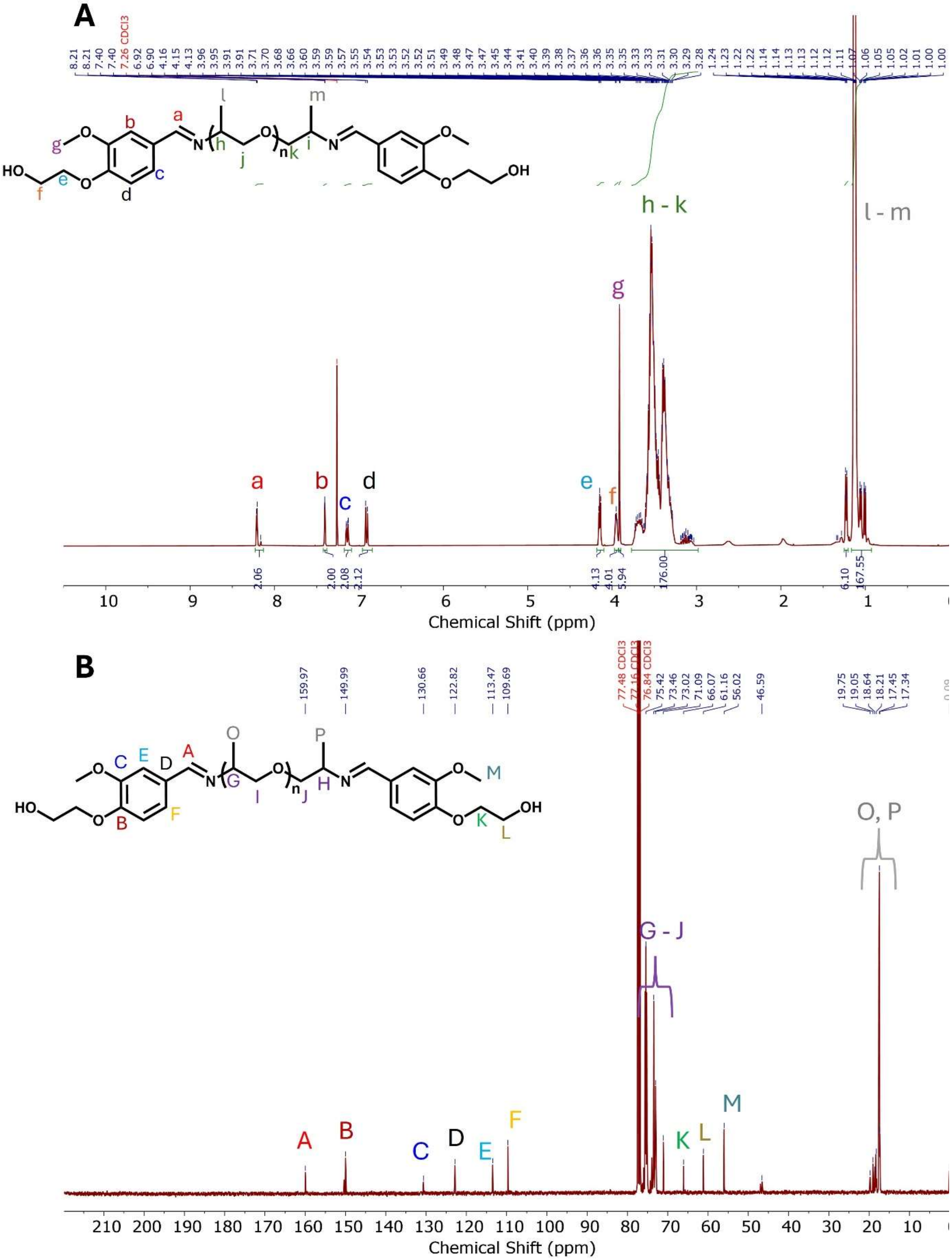

Figure S1. Characterization of imine polyol, **ImP**, via ^{1}H NMR (A) and ^{13}C NMR (B) in $CDCl_3$.

2 . Synthesis and characterization of prepolymers and cured networks

Scheme S1. Curing principle of imine-polyol incorporated 1K-PU system.

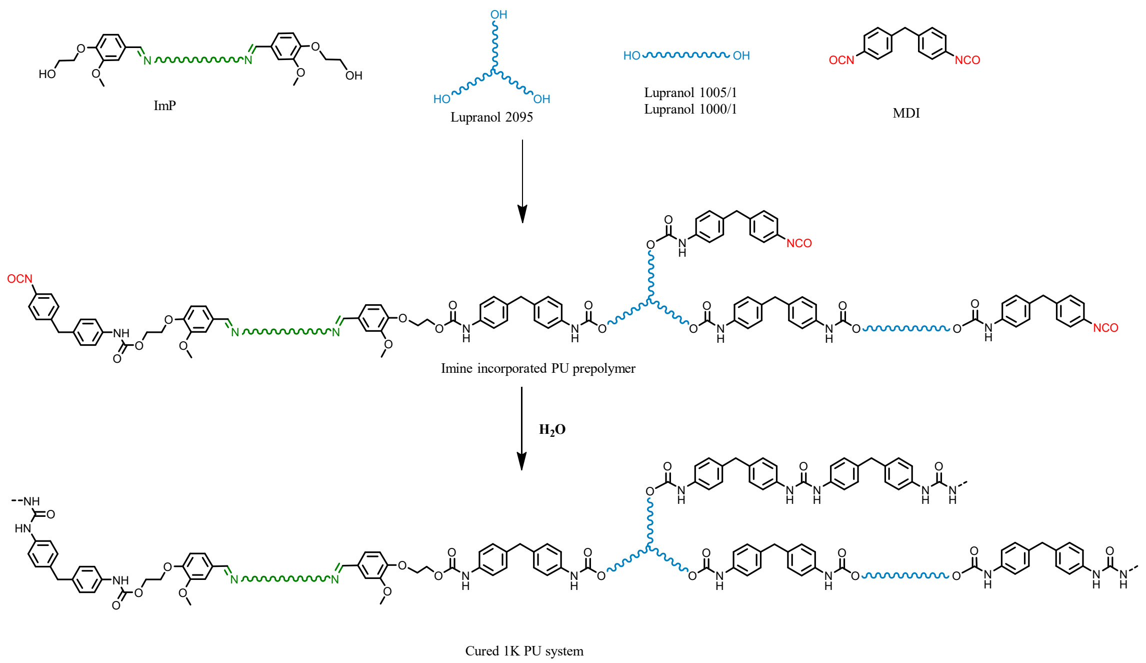

Scheme S2. Acidic hydrolysis of imine-polyol incorporated 1K-PU system.

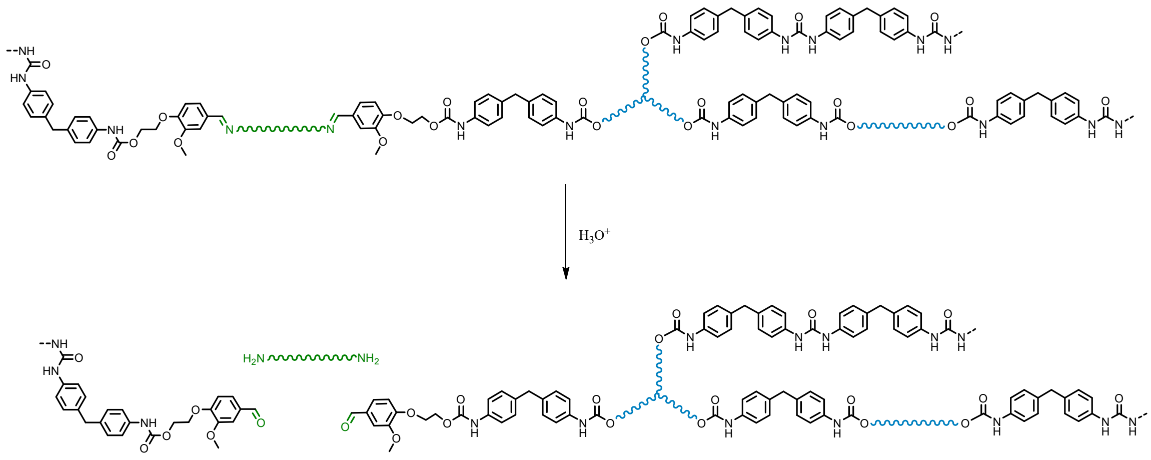

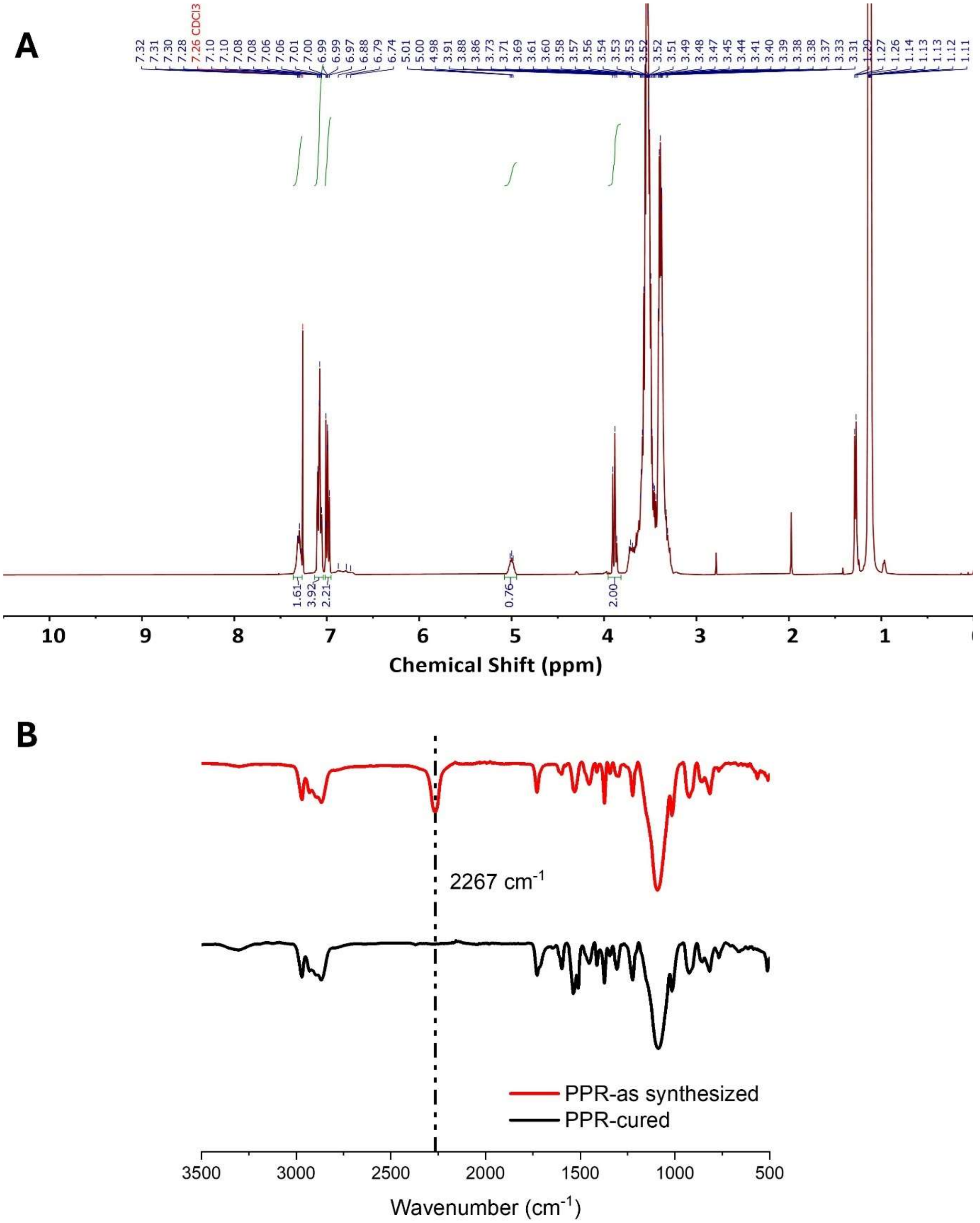

Figure S2. Characterization of **PPR**: ^{1}H NMR spectrum of as-synthesized **PPR** in $CDCl_3$ (A), FTIR spectra of as-synthesized and cured **PPR** (B).

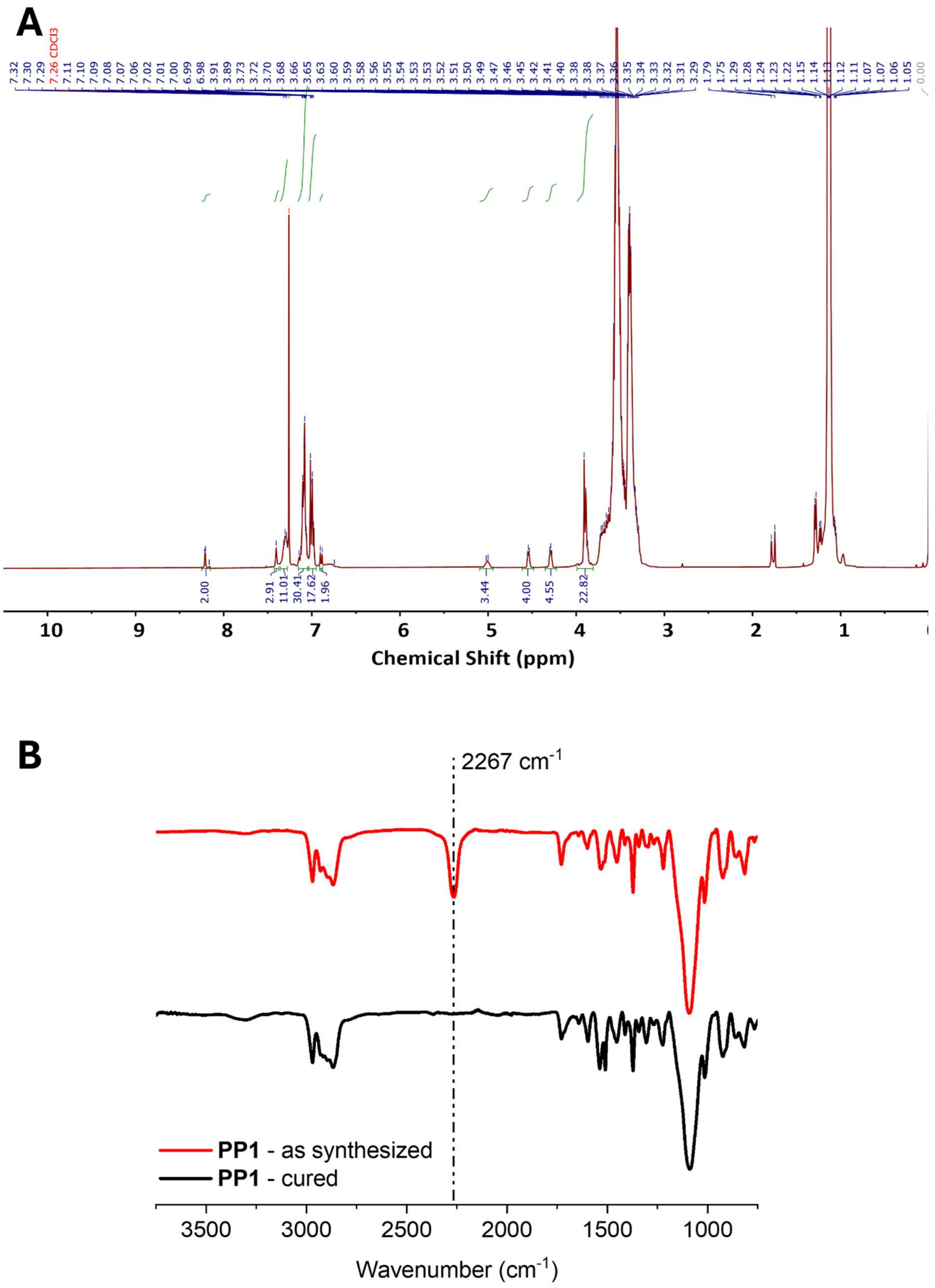

Figure S3. Characterization of **PP1**: ^{1}H NMR spectrum of as-synthesized **PP1** in $CDCl_3$ (A), FTIR spectra of as-synthesized and cured **PP1** (B).

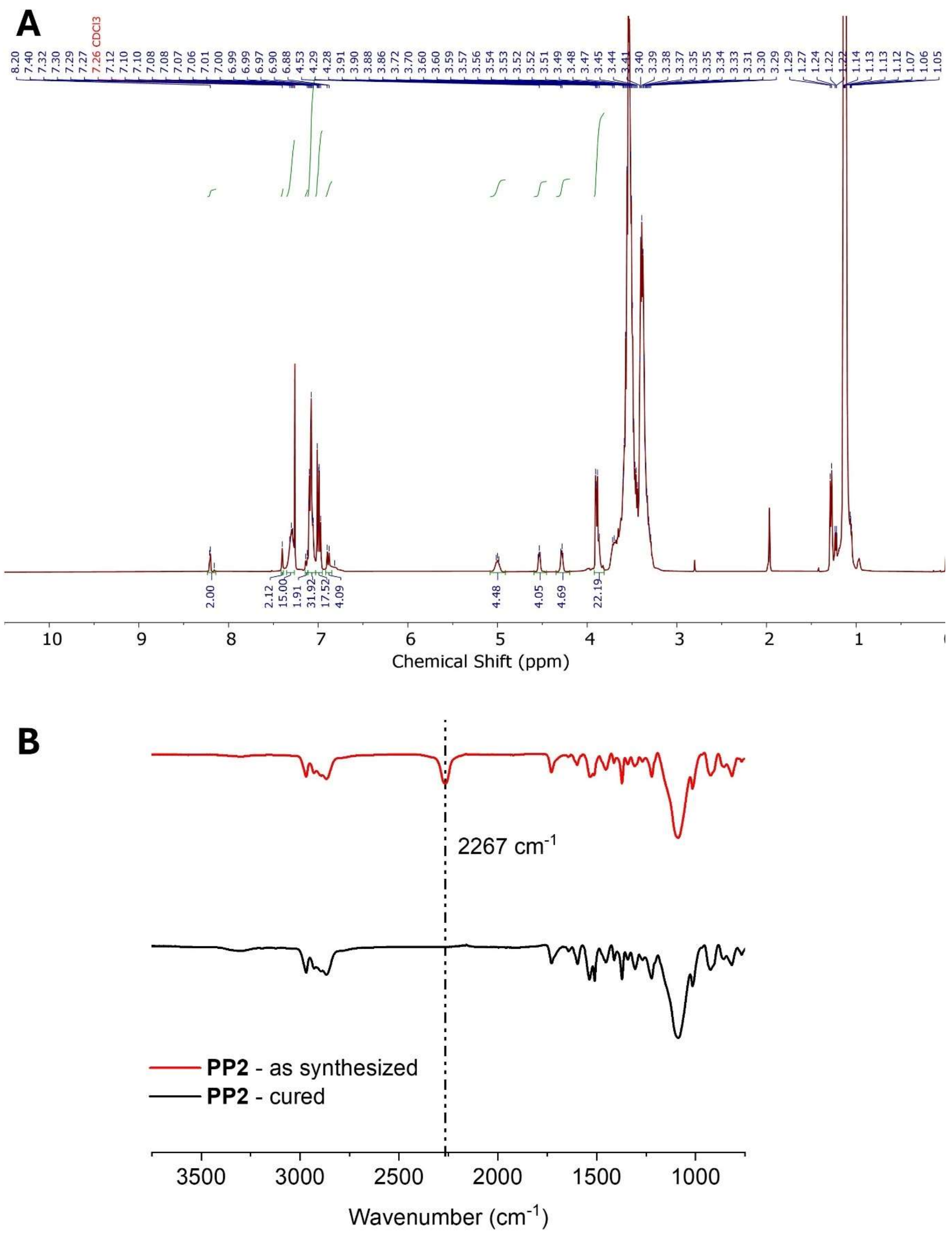

Figure S4. Characterization of **PP2**: ^{1}H NMR spectrum of as-synthesized **PP2** in $CDCl_3$ (A), FTIR spectra of as-synthesized and cured **PP2** (B).

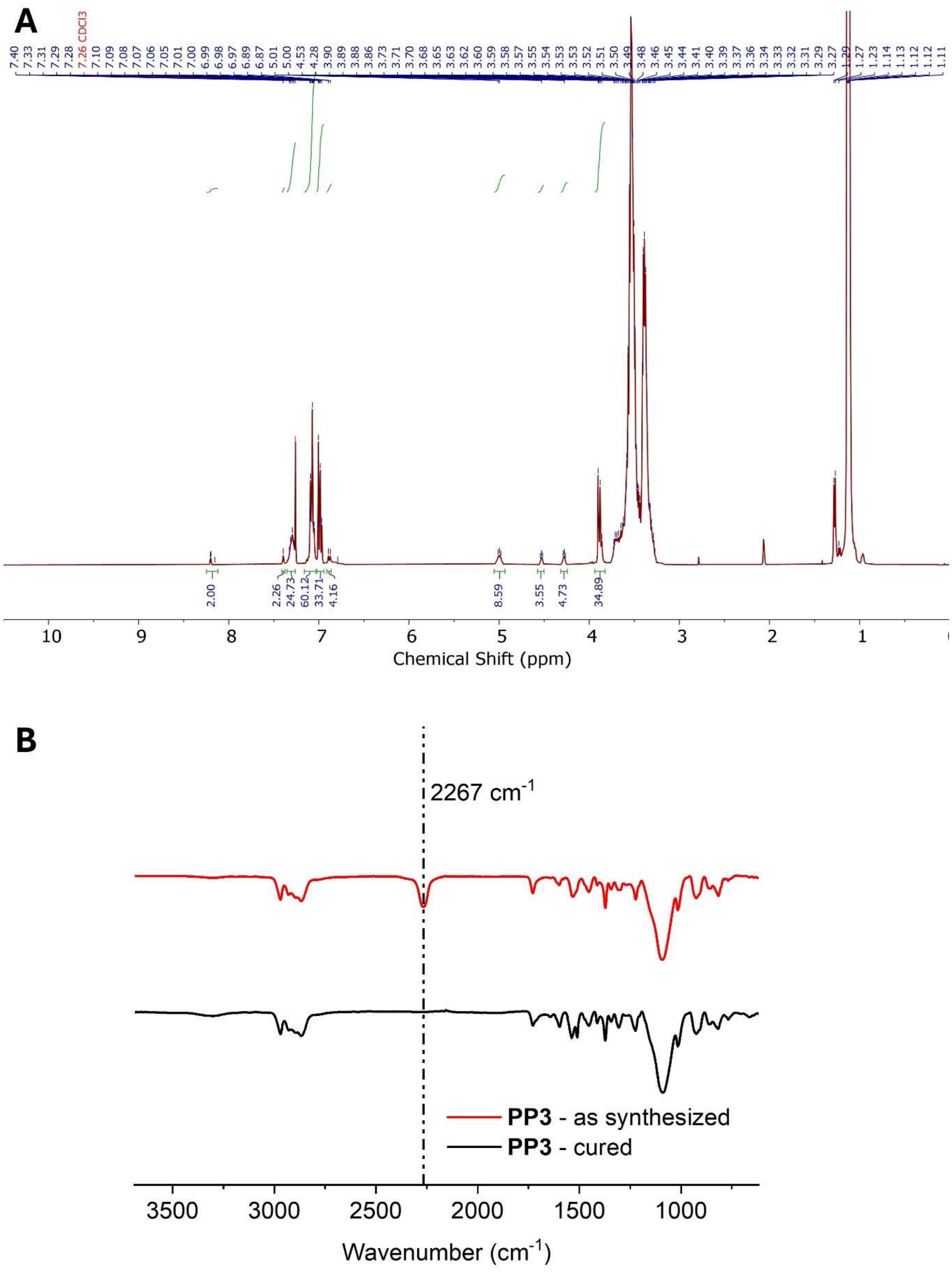

Figure S5. Characterization of **PP3**: ^{1}H NMR spectrum of as-synthesized **PP3** in $CDCl_3$ (A), FTIR spectra of as-synthesized and cured **PP3** (B).

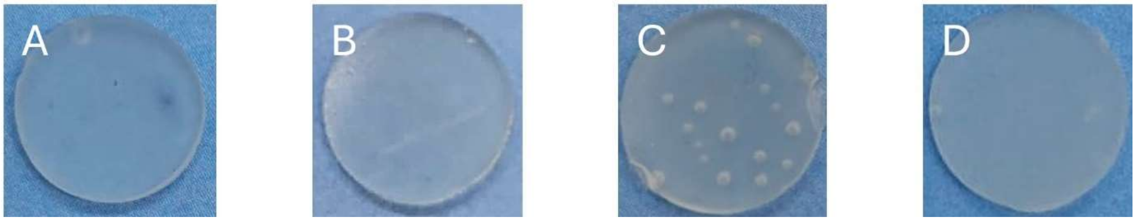

Figure S6. Visual appearances of the cured prepolymers after 1 week of curing at 60% RH: **PPR** (A), **PP1** (B), **PP2** (C) and **PP3** (D). Since the curing process involves the reaction of excess isocyanate with moisture, a carbamic acid intermediate is initially formed. This intermediate is unstable and decomposes into amine end-capped chains and carbon dioxide. While the generated amine end-capped chains cure the prepolymer by reacting with free isocyanates, the released carbon dioxide leads to the formation of pinholes, which is typically observed in 1K PU adhesive systems.[1]

3. Swelling ratio, gel content and crosslink densities

Swelling experiments were performed with THF. The swelling ratio was calculated using equation 1, where q represents the swelling ratio, W_0 the initial weight of polymer, and W_s the weight of swollen network.[2]

$$q = 100x\frac{W_s - W_0}{W_0} \quad (1)$$

Gel fractions were calculated using equation 2, where ϕ stands for gel fraction, W_0 the initial weight of the polymer, and W_1 the weight after drying.[2]

$$\phi = 100x\frac{W_1}{W_0} \quad (2)$$

Crosslinking densities of the networks (ν_e) were calculated using equation 3:[3]

$$\chi V_p^2 + V_p + \ln(1 - V_p) = -\nu_e V_m \left(\sqrt[3]{V_p} - \frac{V_p}{2}\right) \quad (3)$$

where ν_p is the polymer volume fraction, V_m is the molar volume of the solvent (THF = 81.0 mL/mol), χ is the polymer-solvent interaction parameter. [2] The later can be calculated using equation (4): [4]

$$\chi = 0.34 + \frac{V_m}{RT}(\delta_s - \delta_p)^2 \qquad (4)$$

where δ_s and δ_p are the solubility parameters of THF (δ_s = 18.0 $MPa^{0.5}$) and PU polymer, respectively. The solubility parameter of the polymer was taken as that reported for polypropylene glycol (δ_p = 17.5 ± 2.5 $MPa^{0.5}$).[5] χ was taken as 0.34.

Table S1. Swelling ratio, gel content and crosslink densities of the films after complete curing.

	**Swelling Ratio (%)**	**Gel Content (%)**	**Crosslink Density (mol/m^3)**
**PPR**	1369	83.3	18.0
**PP1**	1271	85.6	21.8
**PP2**	1394	82.5	16.1
**PP3**	1376	82.5	17.5

* Swelling ratio, gel content and crosslink densities were calculated after immersing the samples in THF at room temperature for 3 days.

4. Lap-shear tests

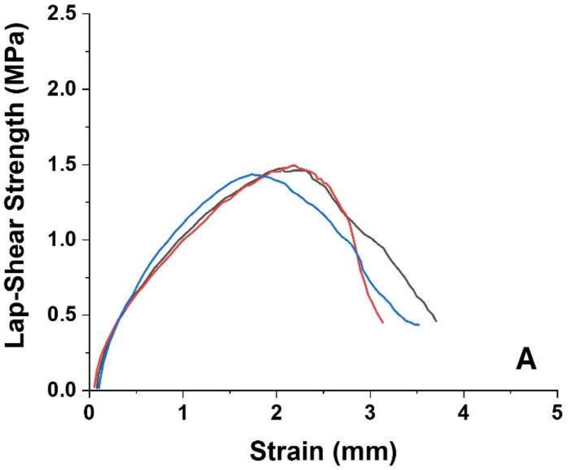

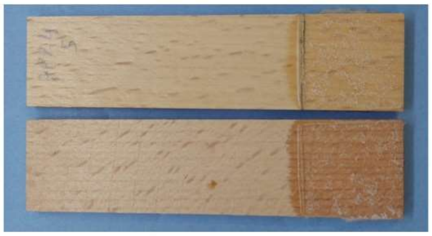

B

Figure S7. Shear stress-strain curves of **PPR**-based adhesive after one week of curing under 60% relative humidity (A). Visual examination of shear surfaces of the adhesive applied (B).

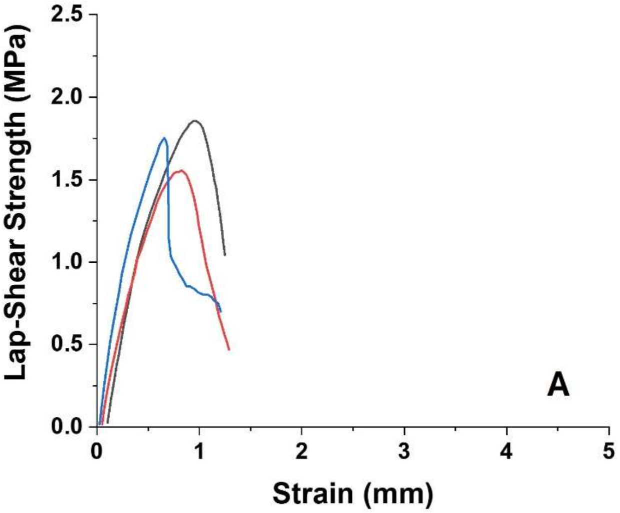

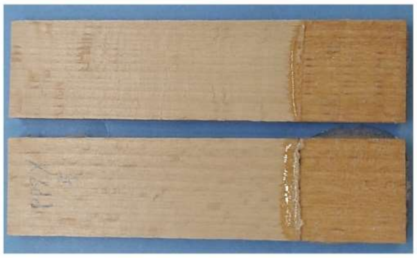

B

Figure S8. Shear stress-strain curves of **PP1**-based adhesive after one week of curing under 60% relative humidity (A). Visual examination of shear surfaces of the adhesive applied (B).

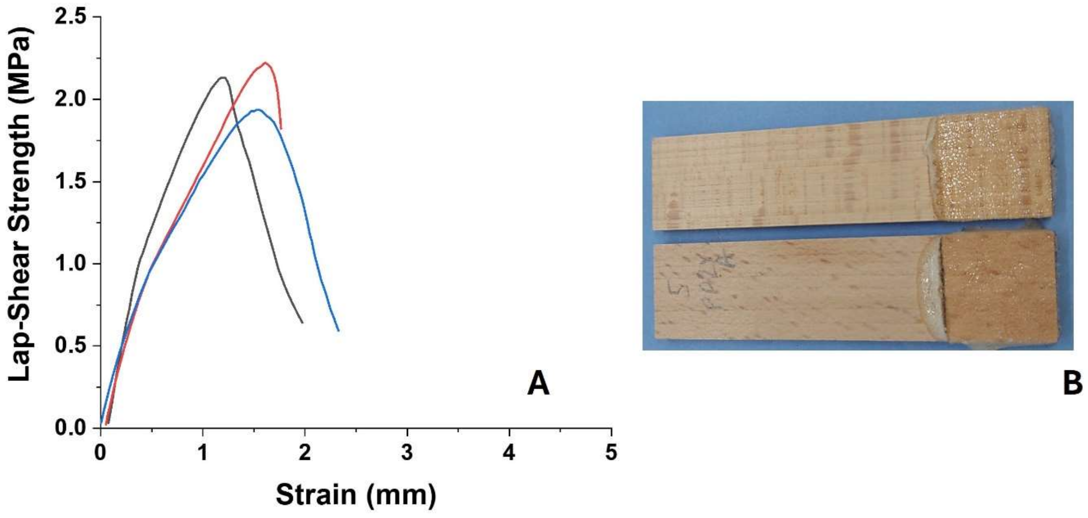

Figure S9. Shear stress-strain curves of **PP2**-based adhesive after one week of curing under 60% relative humidity (A). Visual examination of shear surfaces of the adhesive applied (B).

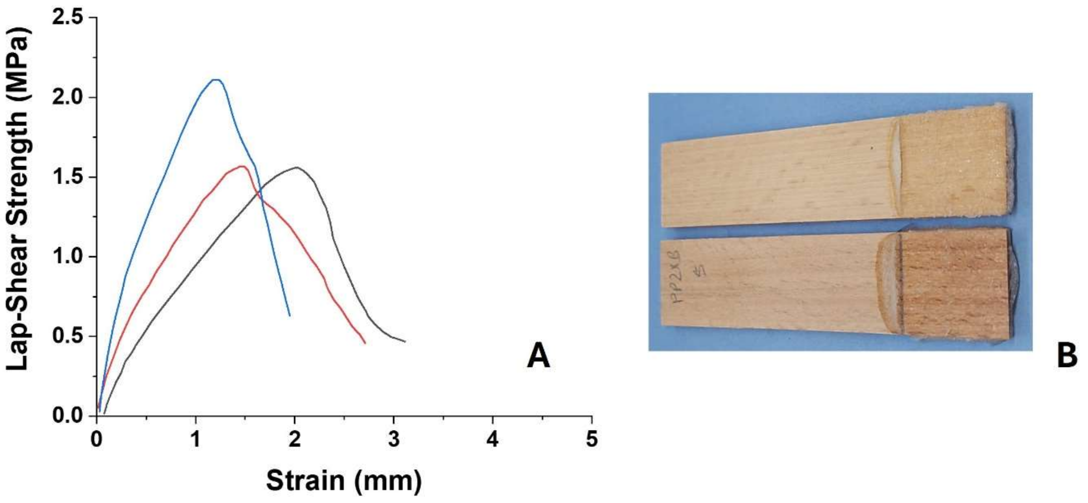

Figure S10. Shear stress-strain curves of **PP3**-based adhesive after one week of curing under 60% relative humidity (A). Visual examination of shear surfaces of the adhesive applied (B).

5. Depolymerization studies

Table S2. Visual assessment of **PPR** films upon exposure to H_2O, 1 M H_3PO_4 (aq) and 1 M citric acid (aq) at 80 °C for 3 and 6 hours.

	**H_2O**	**1 M H_3PO_4 (aq)**	**1 M citric acid (aq)**
**t=0**	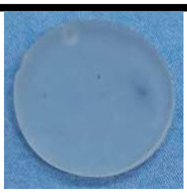	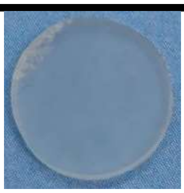	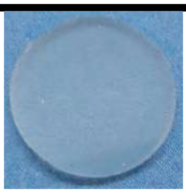
**t=3 h**	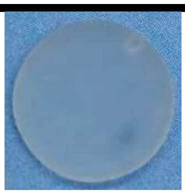	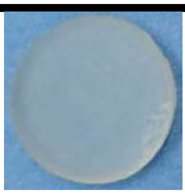	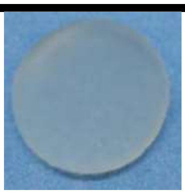
**t=6 h**	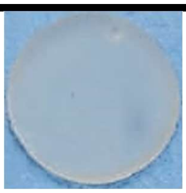	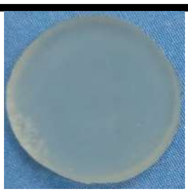	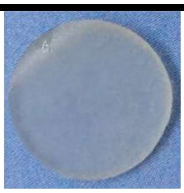

Table S3. Visual assessment of **PP1** films upon exposure to H_2O, 1 M H_3PO_4 (aq) and 1 M citric acid (aq) at 80 °C for 3 and 6 hours.

	**H_2O**	**1 M H_3PO_4 (aq)**	**1 M citric acid (aq)**
**t=0**	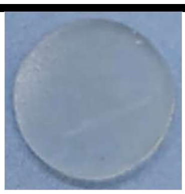	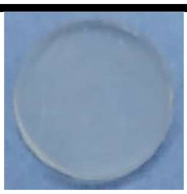	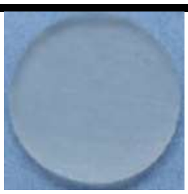
**t=3 h**	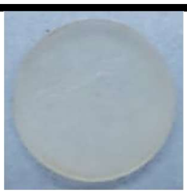	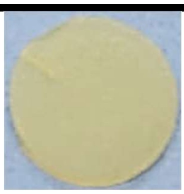	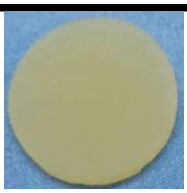
**t=6 h**	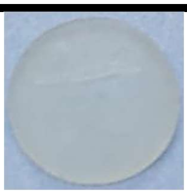	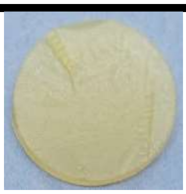	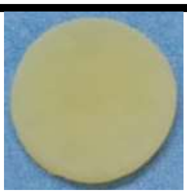

Table S4. Visual assessment of **PP2** films upon exposure to H_2O, 1 M H_3PO_4 (aq) and 1 M citric acid (aq) at 80 °C for 3 and 6 hours.

	**H_2O**	**1 M H_3PO_4 (aq)**	**1 M citric acid (aq)**
**t=0**	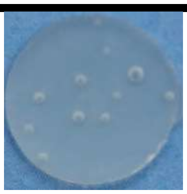	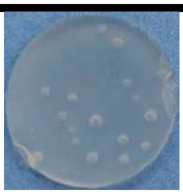	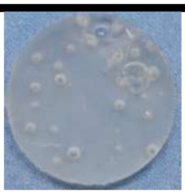
**t=3 h**	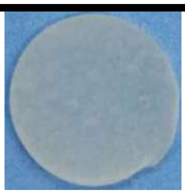	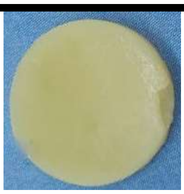	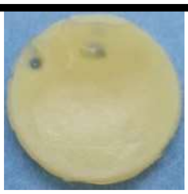
**t=6 h**	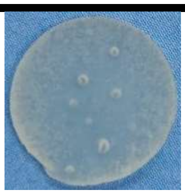	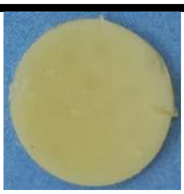	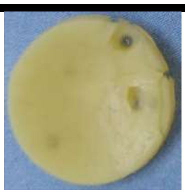

Table S5. Visual assessment of **PP3** films upon exposure to H_2O, 1 M H_3PO_4 (aq) and 1 M citric acid (aq) at 80 °C for 3 and 6 hours.

	**H_2O**	**1 M H_3PO_4 (aq)**	**1 M citric acid (aq)**
**t=0**	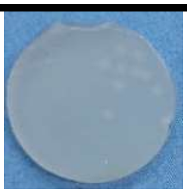	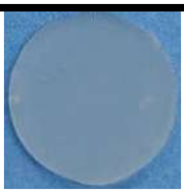	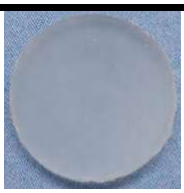
**t=3 h**	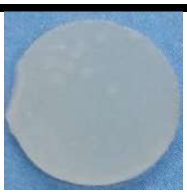	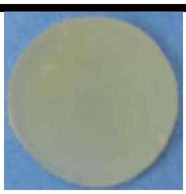	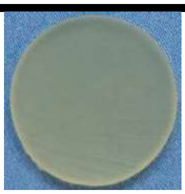
**t=6 h**	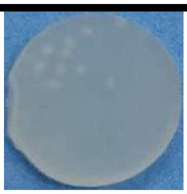	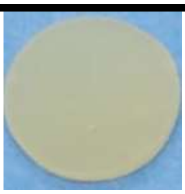	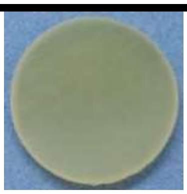

Table S6. Observations after depolymerization studies performed at 80 °C.

Depolymerization Agent	Films			
	PPR	PP1	PP2	PP3
H_2O	–	–	–	–
1 M H_3PO_4	–	Yellowing and weakening	Yellowing and weakening	Yellowing
1 M citric acid	–	Yellowing	Yellowing and weakening	Yellowing

*: Identical conditions were employed for the depolymerization which was performed at 80 °C with a constant stirring rate at 200 rpm. – represents no visual changes after 6 h under specified conditions.

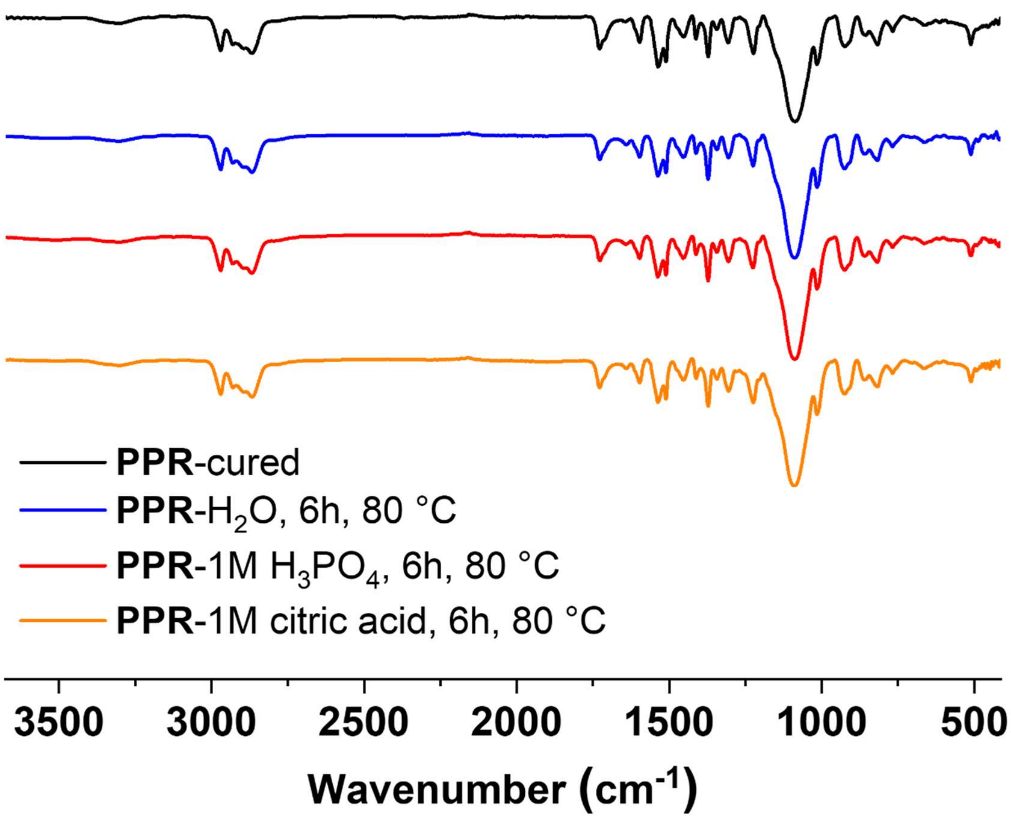

Figure S11. FTIR analysis of **PPR** films upon exposure to H_2O, 1 M H_3PO_4 or 1 M citric acid at 80 °C after 6 hours.

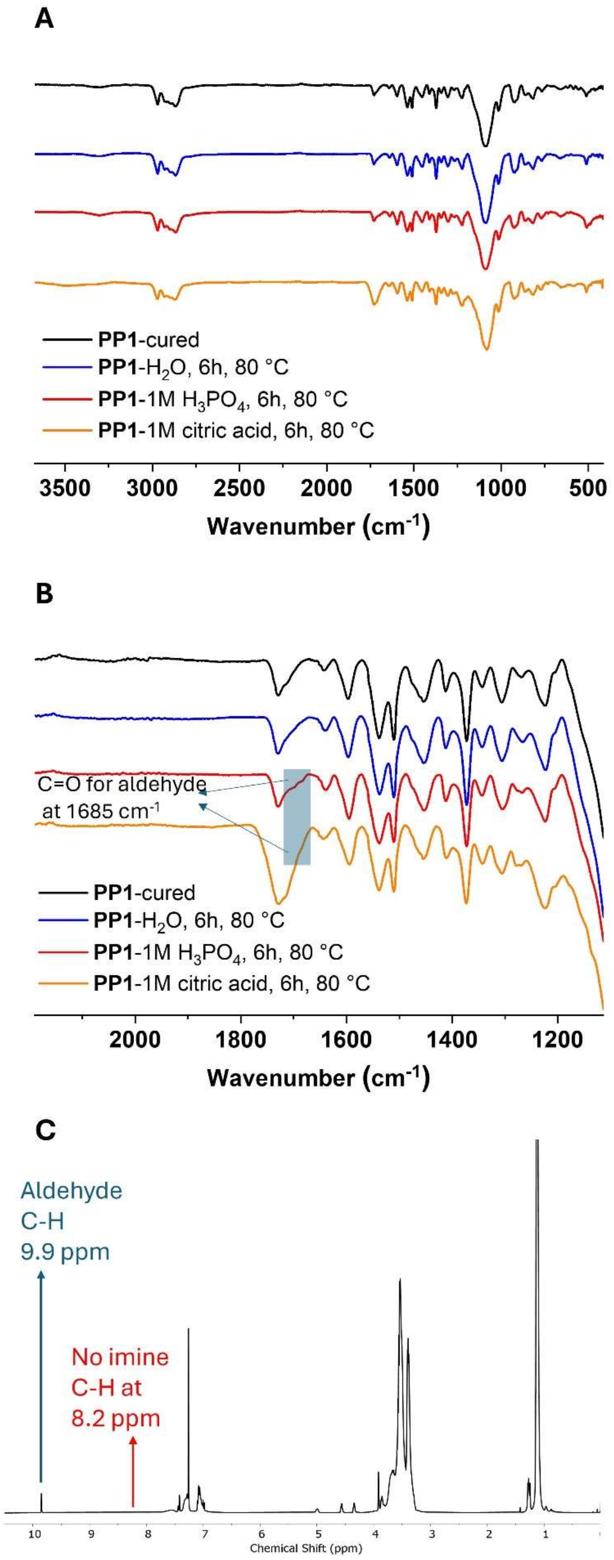

Figure S12. FTIR analysis of **PP1** films upon exposure to H_2O, 1 M H_3PO_4 or 1 M citric acid at 80 °C after 6 hours (A), with zoom-in image (B). ^1H NMR ($CDCl_3$) of the of **PP1** films upon exposure to 1 M H_3PO_4 at 80 °C after 6 hours (C).

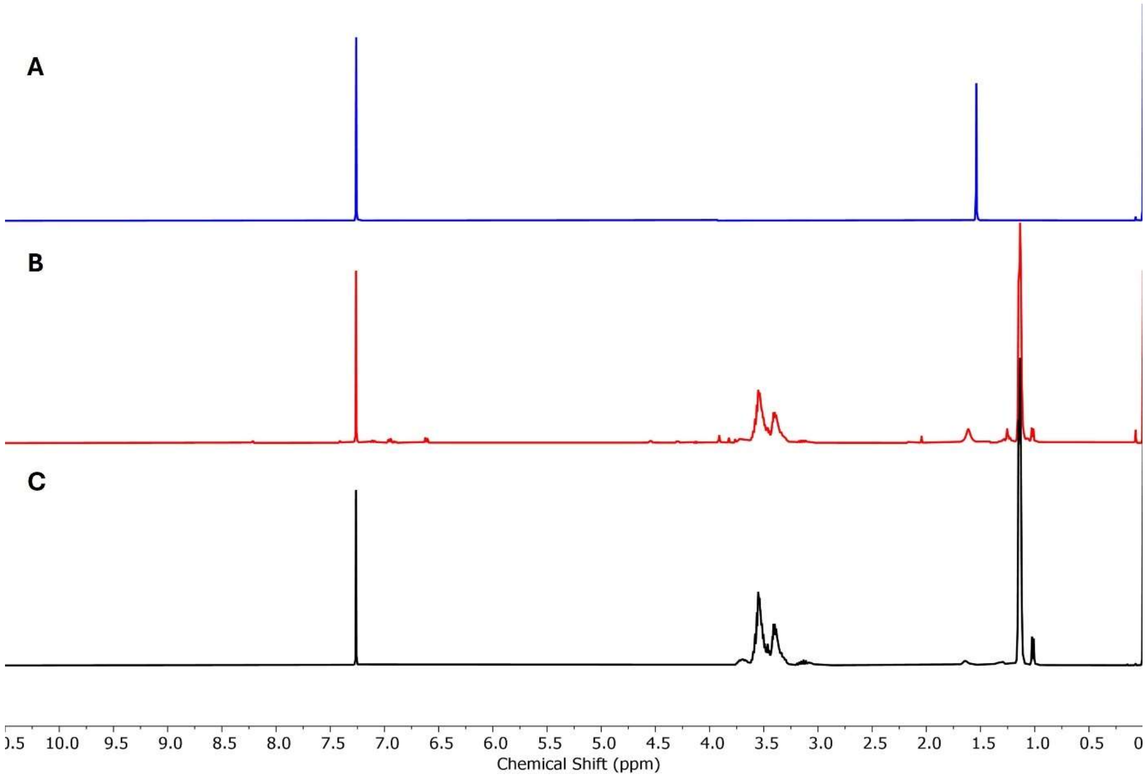

Figure S13. ^{1}H NMR analysis of the aqueous phase of the **PP1** film upon exposure to H_2O at 80 °C after 6 hours (A), the aqueous phase of the **PP1** film upon exposure to 1 M H_3PO_4 at 80 °C after 6 hours (B), Jeffamine® D2000 (C) in $CDCl_3$.

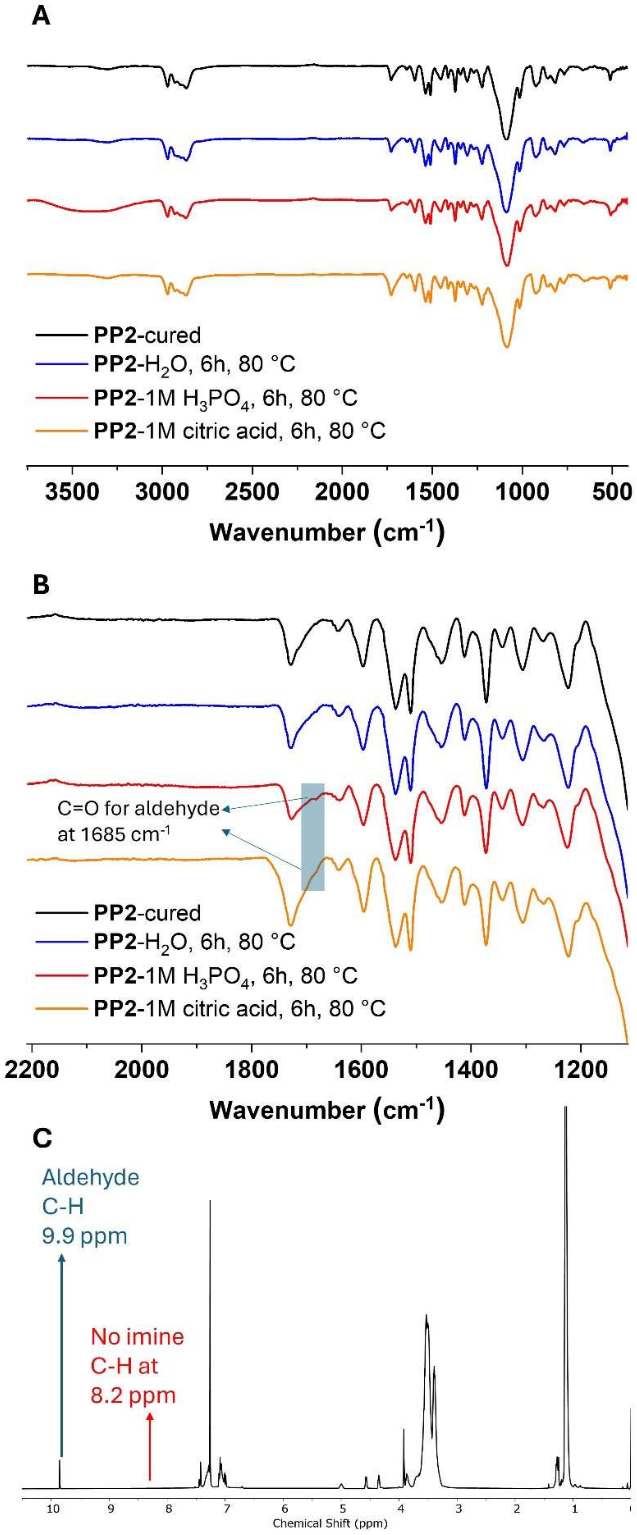

Figure S14. FTIR analysis of **PP2** films upon exposure to H_2O, 1 M H_3PO_4 or 1 M citric acid at 80 °C after 6 hours (A), with zoom-in image (B). ^1H NMR ($CDCl_3$) of the of **PP2** films upon exposure to 1 M H_3PO_4 at 80 °C after 6 hours (C).

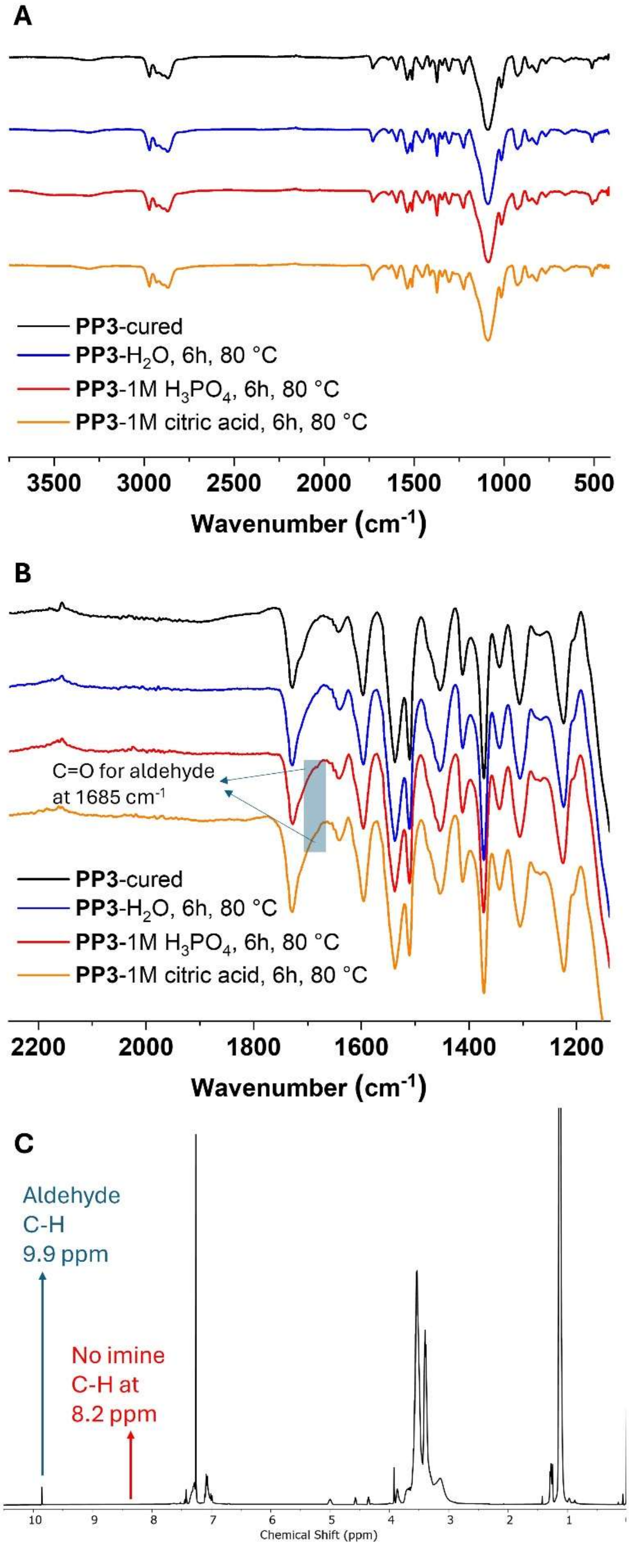

Figure S15. FTIR analysis of **PP3** films upon exposure to H_2O, 1 M H_3PO_4 or 1 M citric acid at 80 °C after 6 hours (A), with zoom-in image (B). ^1H NMR ($CDCl_3$) of the of **PP3** films upon exposure to 1 M H_3PO_4 at 80 °C after 6 hours (C).

6. Debonding studies

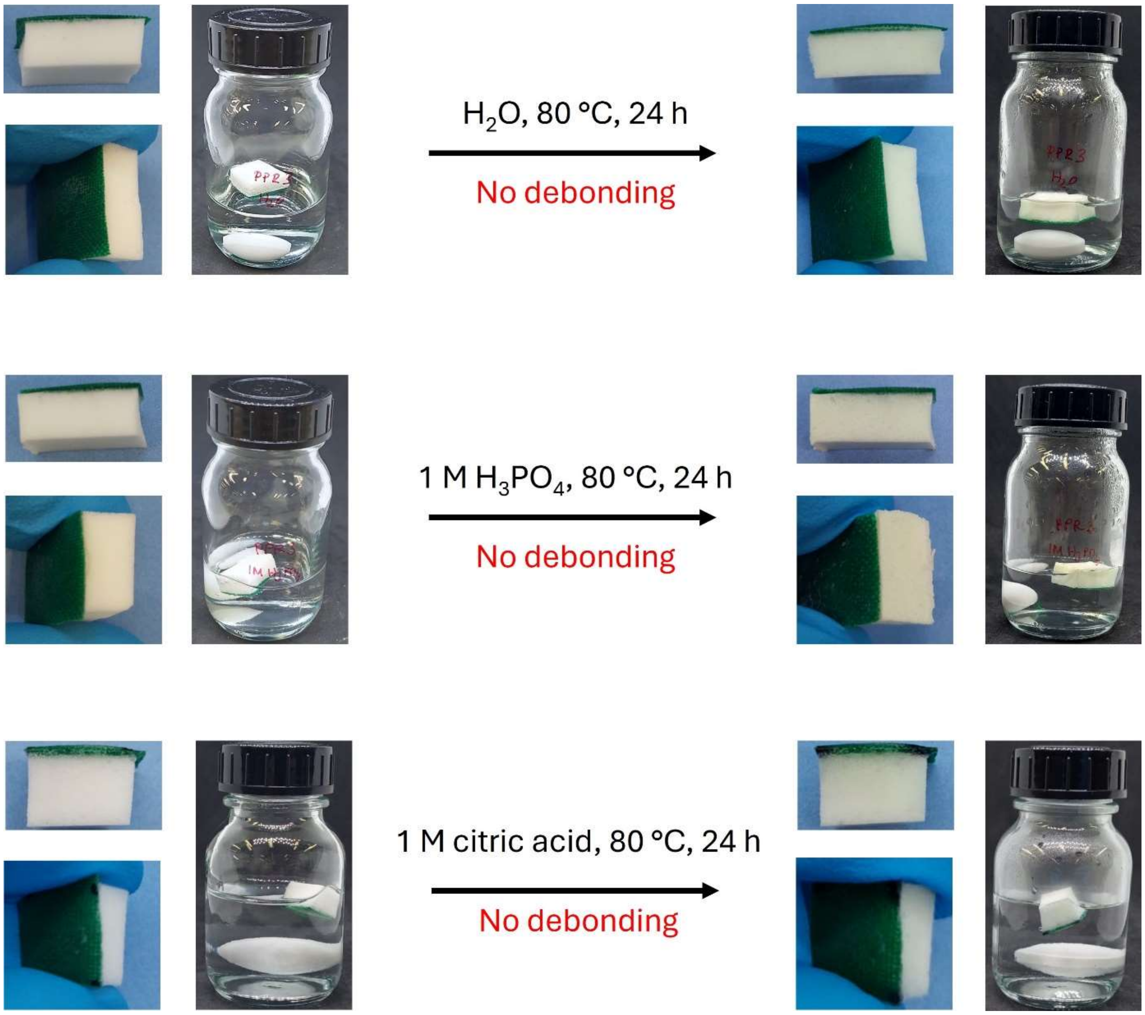

Figure S16. Debonding studies conducted on the **PPR**-based adhesive which was applied between PU foam and PET textile in H_2O, 1 M H_3PO_4, and 1 M citric acid at 80 °C.

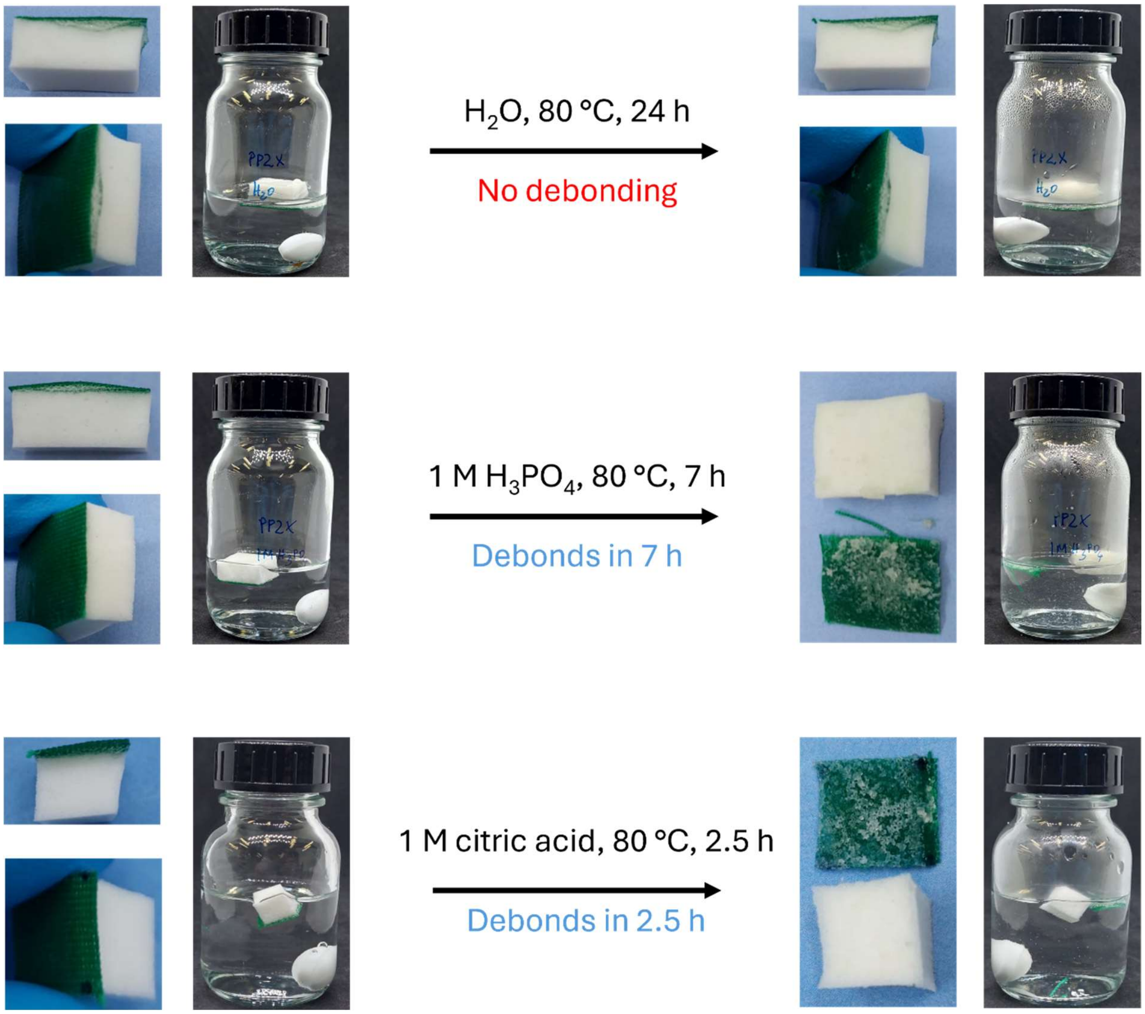

Figure S17. Debonding studies conducted on the **PP1**-based adhesive which was applied between PU foam and PET textile in H_2O, 1 M H_3PO_4, and 1 M citric acid at 80 °C.

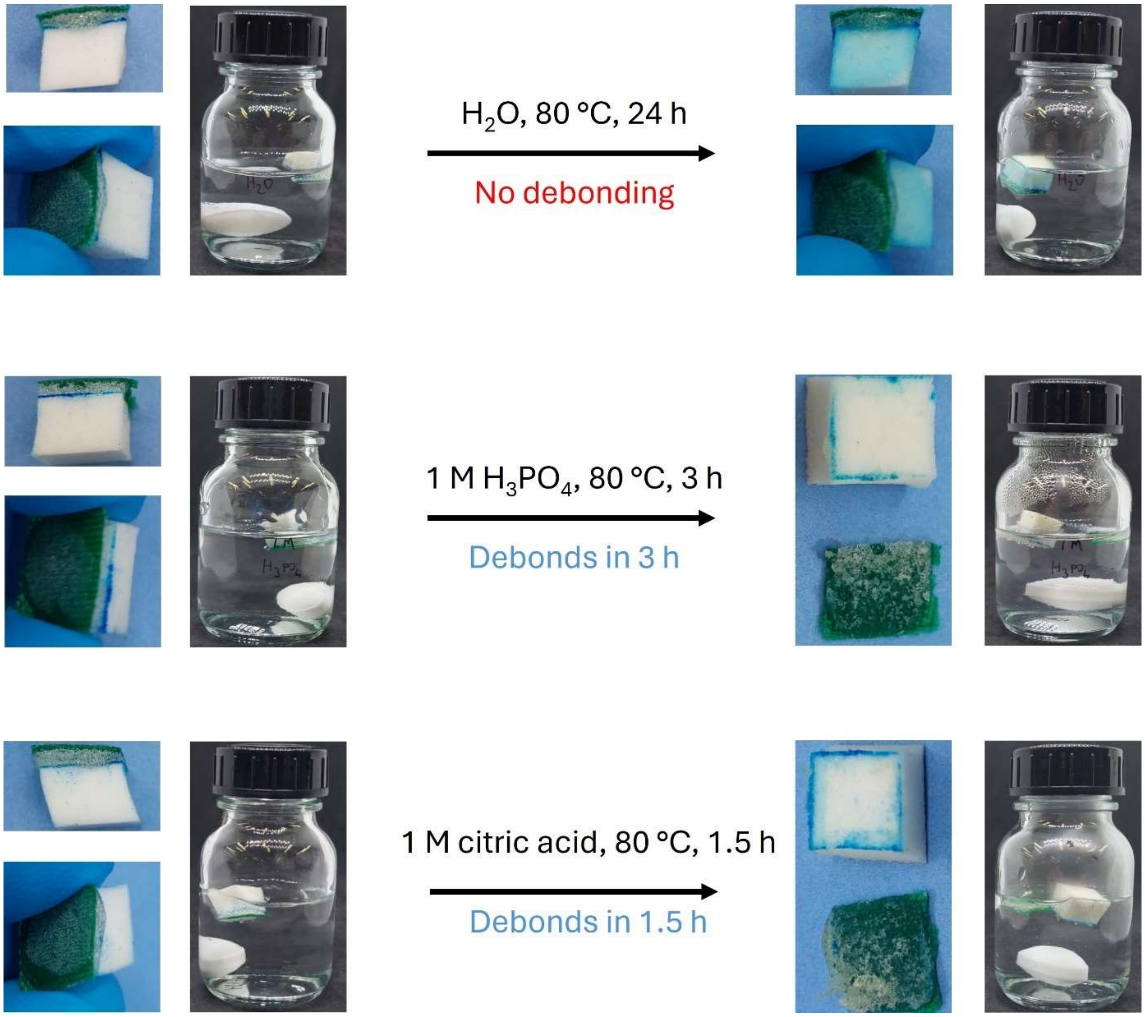

Figure S18. Debonding studies conducted on the **PP2**-based adhesive which was applied between PU foam and PET textile in H_2O, 1 M H_3PO_4, and 1 M citric acid at 80 °C.

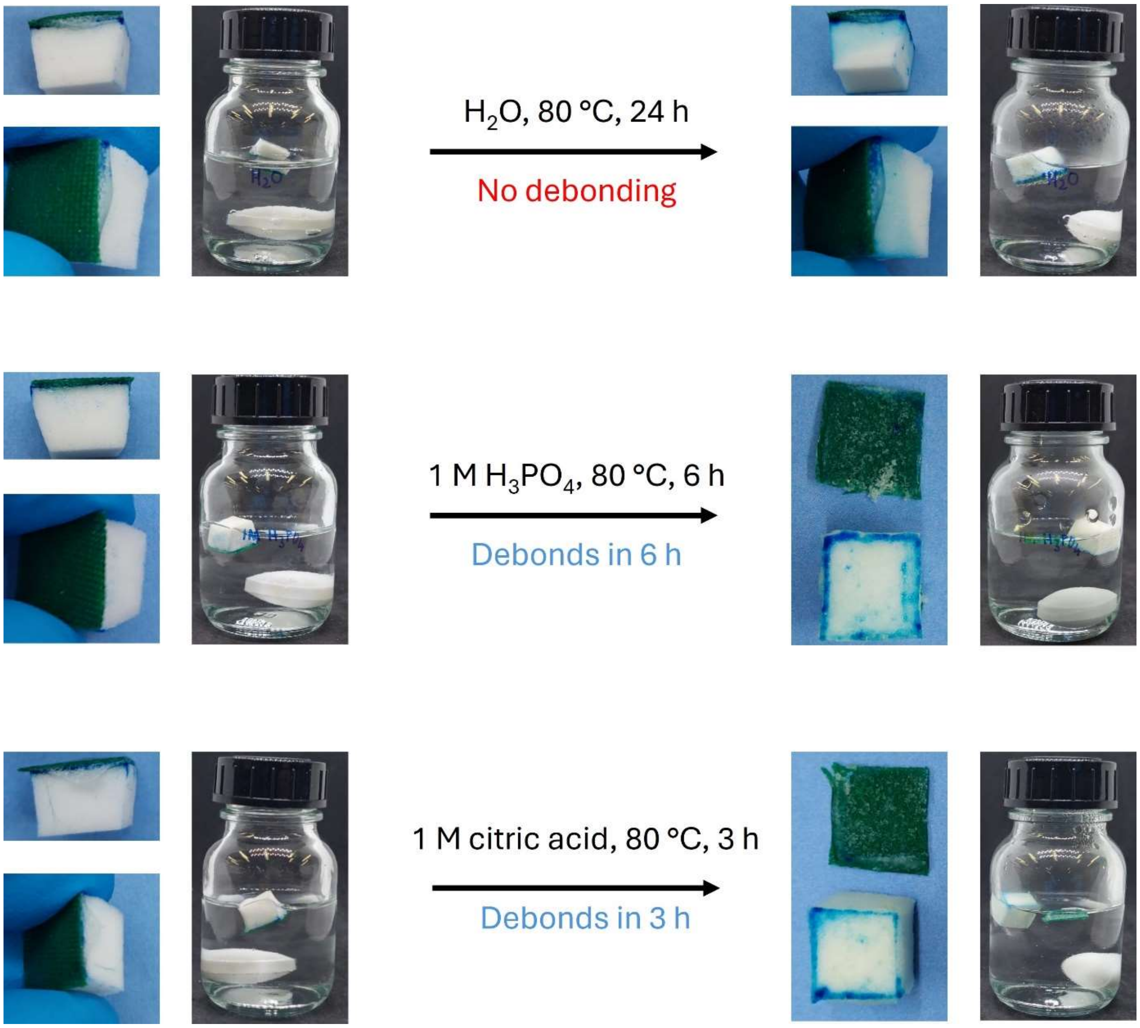

Figure S19. Debonding studies conducted on the **PP3**-based adhesive which was applied between PU foam and PET textile in H_2O, 1 M H_3PO_4, and 1 M citric acid at 80 °C.

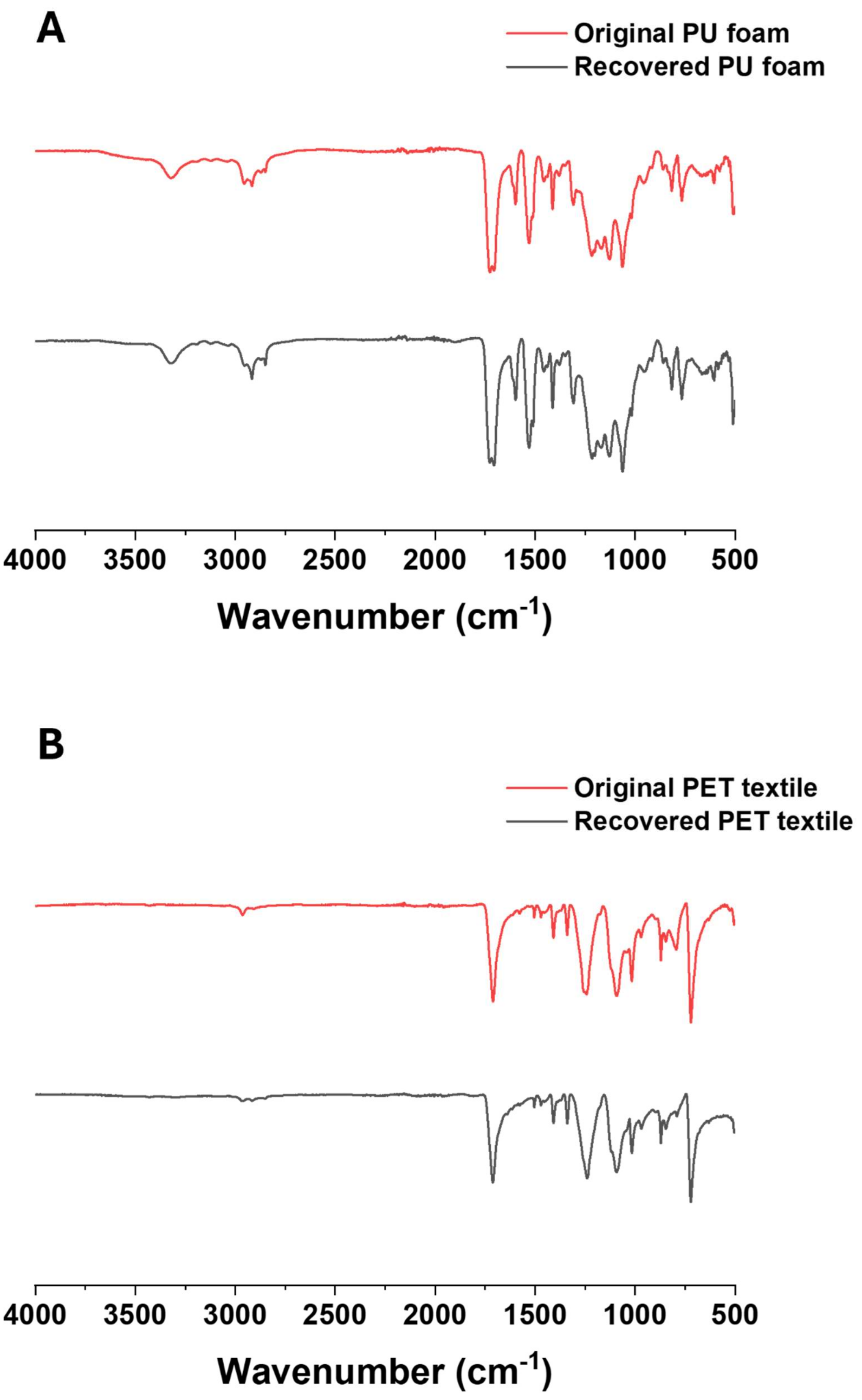

Figure S20. FTIR analysis of substrates bonded with **PP1** after debonding with 1 M H_3PO_4 at 80 °C, compared with their original FTIR spectra: PU foam (A), PET textile (B).

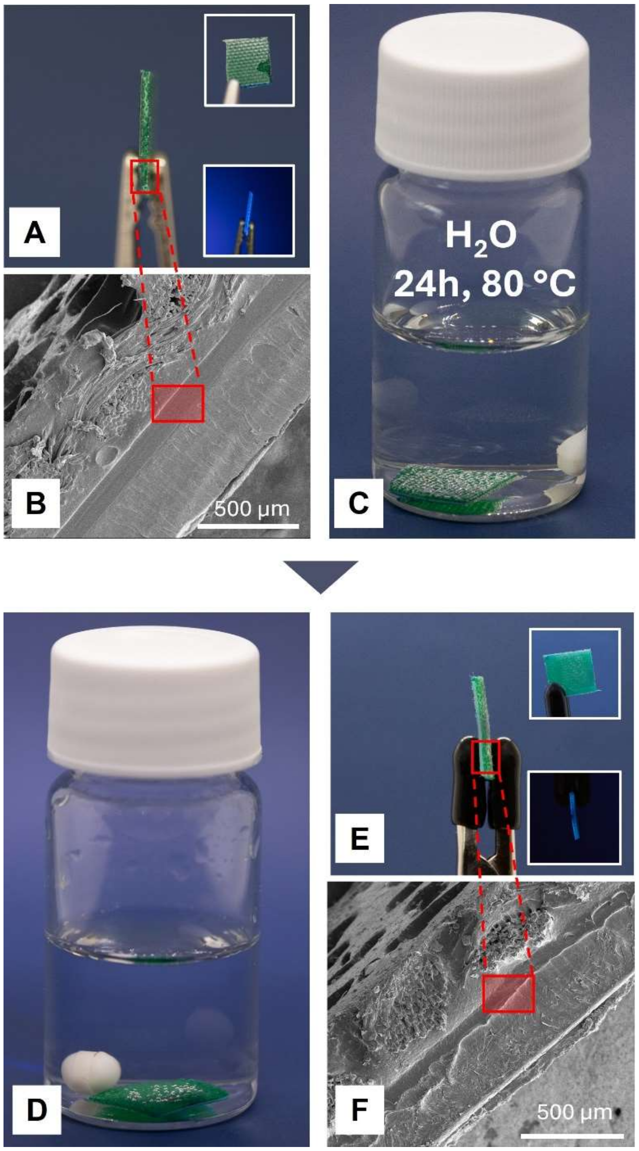

Figure S21. Debonding studies conducted in water at 80 °C on the **PP3**-based adhesive, which was applied between PET foil and PET textile: the images of the bonded substrates before water exposure from the interface and the top (inset-up) under normal light and under UV light illumination (inset-bottom) (A), SEM micrograph of untreated adhesion interface of the PET foil and PET textile, where the scale bar is 500 µm (B), the image of the bonded substrates in water at t=0 (C), the image of the treated substrates in water after 24 h (D), the image of the treated substrates from the interface and the top (inset-up) under normal light and under UV light illumination (inset-bottom) after 24 h (E), SEM micrograph of the treated adhesion interface of the PET foil and PET textile, where the scale bar is 500 µm (F).

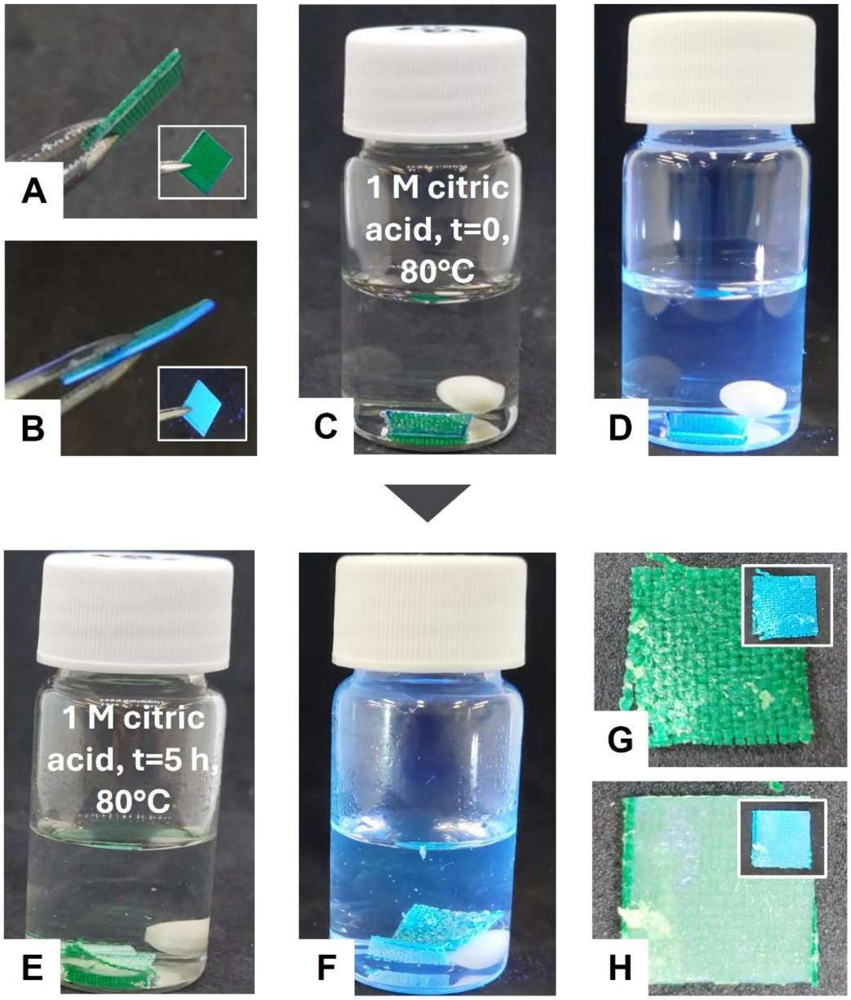

Figure S22. Debonding studies conducted in 1 M citric acid at 80 °C on the **PP3**-based adhesive which was applied between PET foil and PET textile: the images of the bonded substrates before citric acid exposure from the interface and the top (inset) (A), the images of the bonded substrates before citric acid exposure from the interface and the top (inset) under UV light illumination (B), the image of the bonded substrates in citric acid solution at t=0 under normal light (C) and under 365 nm UV light illumination (D), the image of the debonded substrates in citric acid solution at t=5 h under normal light (E) and under 365 nm UV light illumination (F), the image of the debonded PET textile under normal light and under 365 nm UV light (inset) illumination (G), the image of the debonded PET foil under normal light and under 365 nm UV light (inset) illumination (H).

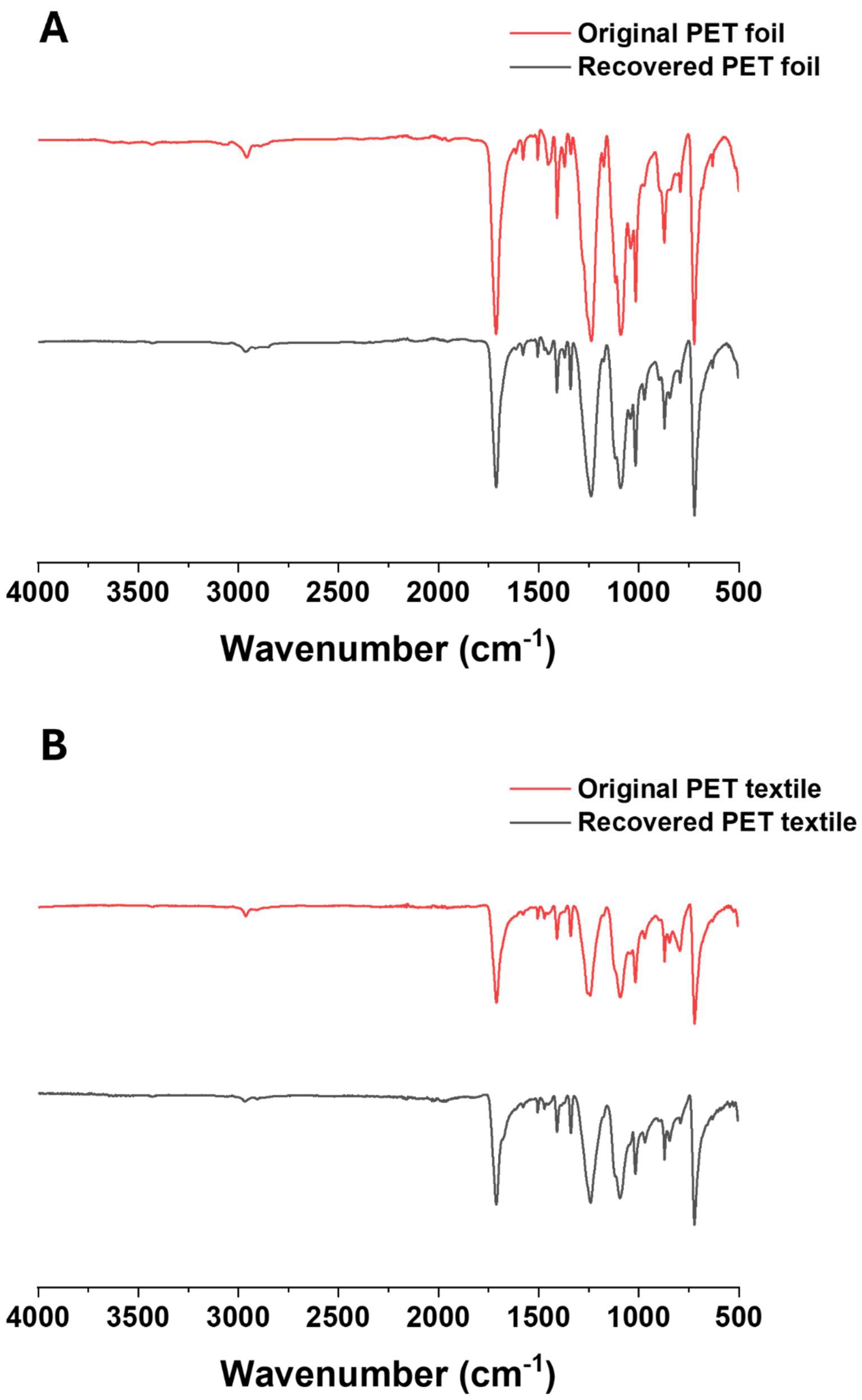

Figure S23. FTIR analysis of substrates bonded with **PP3** after debonding with 1 M H_3PO_4 at 80 °C, compared with their original FTIR spectra: PET foil (A), PET textile (B).

Table S7. Debonding conditions and time required for the debonding of adhesives between different substrates.

Substrate combination	Debonding Agent	Adhesives			
		PPR	PP1	PP2	PP3
PU foam - PET textile	H_2O	–	–	–	–
	1 M H_3PO_4	–	7 h	3 h	6 h
	1 M citric acid	–	2.5 h	1.5 h	3.5 h
PET foil - PET textile	H_2O	n.t.	n.t.	n.t.	–
	1 M H_3PO_4	n.t.	n.t.	n.t.	3 h
	1 M citric acid	n.t.	n.t.	n.t.	5 h

*: Identical conditions were employed for the debonding which was performed at 80 °C with a constant stirring rate at 200 rpm. – represents no debonding after 24 h under specified conditions while n.t. stands for "not tested".

Table S8. Comparison of Debonding Methods

Substrate	Stimuli	Debonding Chemistry	Conditions	Base Polymer	Reference
Wafer	Thermal	retro-Diels-Alder	200 °C	Acrylic copolymer	**6**
Stainless steel	Thermal	retro-Diels-Alder	120 °C	Polyurethane	**7**
Aluminum	Thermal	retro-Diels-Alder	150 °C, 2 h	Epoxy	**8**
Leather/SBR rubber	Thermal	retro-Diels-Alder	110 °C, 2 h	Polyurethane	**9**
Stainless steel	Thermal	Oxime	Heating with hair dryer, 5 min	Polyurethane	**10**
Wood	Thermal	Thermally expandable particles and retro-Diels Alder	150 °C	Polyurethane	**11**
Glass	Thermal, magnetic	Magnetic Fe_3O_4 particles	Heat, 30 s – 5 min	Polyurethane	**12**
Quartz	Light	Dimerization [2 + 2] cycloaddition	254 nm light + weight, 5 min	Siloxane backbone with coumarin groups	**13**
Quartz	Light	Dimerization[4 + 4] cycloaddition	254 nm light, 20 h	Anthracene-based epoxy resin	**14**
Quartz	Light	Azobenzene isomerization	365 nm UV light, 15 min	Acrylate polymers	**15**

Substrate	Stimuli	Debonding Chemistry	Conditions	Base Polymer	Reference
Glass	Light	Isomerization (open and closed ring)	525 nm VIS light, 6 h	Spiropyran polymers	**16**
Aluminum	Chemical	Depolymerization of backbone and reduction in crosslink density	0.025 M TBAF/acetonitrile, 40 °C, 3 h	Polyurethane	**17**
PU foam/PET textile; PET foil/PET textile; wood	Chemical	Depolymerization of backbone and reduction in crosslink density by imine hydrolysis	1 M H_3PO_4 or 1 M citric acid, 80 °C, 1.5 – 7 h	Polyurethane	**Our work**

Our work offers a broader selection of substrates compared to other debonding methods. Additionally, debonding can be easily achieved using environmentally friendly, mild acids.

References

1) Türel, T.; Eling, B.; Cristadoro, A.; Mathieu, T.; Linnenbrink, M.; Tomović, Ž. Novel Furfural-Derived Polyaldimines as Latent Hardeners for Polyurethane Adhesives. *ACS Appl. Mater. Interfaces* **2024**, 16, 6414-6423. DOI: 10.1021/acsami.3c17416.

2) Song, R.-H.; Liu, Z.; Geng, X.; Ye, L.; Zhang, A.; Feng, Z. Preparation and Characterization of Cross-Linked Polyurethanes Using β-CD [3]PR as Slide-Ring Cross-Linker. *Polymer* **2022**, *249*, 124862. DOI: 10.1016/j.polymer.2022.124862.

3) Flory, P. J.; Rehner, J. Statistical Mechanics of Cross-Linked Polymer Networks II. Swelling. *J. Chem. Phys.* **1943**, *11*, 521–526. DOI: 10.1063/1.1723792.

4) Bristow, G. M.; Watson, W. F. Cohesive Energy Densities of Polymers. Part 1.—Cohesive Energy Densities of Rubbers by Swelling Measurements. *Trans. Faraday Soc.* **1958**, *54*, 1731–1741. DOI: 10.1039/tf9585401731.

5) Van Krevelen, D. W.; Nijenhuis, K. T. Properties of Polymers: Their Correlation with Chemical Structure; Their Numerical Estimation and Prediction from Additive Group Contributions; Elsevier Science, **2009**. ISBN: 978-0-08-054819-7.

6) Wouters, M.; Burghoorn, M.; Ingenhut, B. L. J.; Timmer, K.; Rentrop, C.; Bots, T. L.; Oosterhuis, G. J. E.; Fischer, H. Tuneable adhesion through novel binder technologies. Prog. Org. Coat. 2011, 72, 152–158. DOI: 10.1016/j.porgcoat.2010.12.014.

7) Wu, M.; Liu, Y.; Du, P.; Wang, X.; Yang, B. Polyurethane hot melt adhesive based on Diels-Alder reaction. Int. J. Adhes. Adhes. 2020, 100, 102597. DOI: 10.1016/j.ijadhadh.2020.102597.

8) Ramimoghadam, D.; Szmalko, D.; Dilag, J.; Ladani, R. B.; Mouritz, A. P.; Bateman, S. Thermally reversible prototype adhesive via the furan–maleimide Diels–Alder reaction. *Int. J. Adhes. Adhes.* **2024**, *128*, 103522. DOI: 10.1016/j.ijadhadh.2023.103522.

9) Carbonell-Blasco, M. P.; Moyano, M. A.; Hernández-Fernández, C.; Sierra-Molero, F. J.; Pastor, I. M.; Alonso, D. A.; Arán-AíS, F.; Orgilés-Calpena, E. Polyurethane Adhesives with Chemically Debondable Properties via Diels–Alder Bonds. *Polymers* **2023**, 16, 21. DOI: 10.3390/polym16010021.

10) Zhong, K.; Guan, Q.; Sun, W.; Qin, M.; Liu, Z.; Zhang, L.; Xu, J.; Zhang, F.; You, Z. Hot-Melt adhesive based on dynamic Oxime–Carbamate bonds. *Ind. Eng. Chem. Res.* **2021**, *60*, 6925–6931. DOI: 10.1021/acs.iecr.1c00768

11) Thoma, J. L.; Elsener, R.; Burgert, I.; Schubert, M. Chemical and Physical Debonding-on-Demand of Poly(urethane urea) Thermoset Adhesives to Facilitate the Recycling of Engineered Wooden Products. *ACS Appl. Polym. Mater.* **2024**, *6*, 5778–5787. DOI: 10.1021/acsapm.4c00439.

12) Salimi, S.; Babra, T. S.; Dines, G. S.; Baskerville, S. W.; Hayes, W.; Greenland, B. W. Composite polyurethane adhesives that debond-on-demand by hysteresis heating in an oscillating magnetic field. *Eur. Polym. J.* **2019**, *121*, 109264.

13) Inada, M.; Horii, T.; Fujie, T.; Nakanishi, T.; Asahi, T.; Saito, K. Debonding-on-demand adhesives based on photo-reversible cycloaddition reactions. *Mater. Adv.* **2023**, *4* (5), 1289–1296. DOI: 10.1039/d2ma01048h.

14) Liu, Z.; Cheng, J.; Zhang, J. An Efficiently Reworkable Thermosetting Adhesive Based on Photoreversible [4+4] Cycloaddition Reaction of Epoxy-Based Prepolymer with Four Anthracene End Groups. *Macromol. Chem. Phys.* **2020**, *222,* 2000298. DOI: 10.1002/macp.202000298.

15) Zhou, Y.; Chen, M.; Ban, Q.; Zhang, Z.; Shuang, S.; Koynov, K.; Butt, H.-J.; Kong, J.; Wu, S. Light-Switchable polymer adhesive based on photoinduced reversible Solid-to-Liquid transitions. *ACS Macro Lett.* **2019**, 8, 968–972. DOI: 10.1021/acsmacrolett.9b00459.

16) Imato, K.; Momota, K.; Kaneda, N.; Imae, I.; Ooyama, Y. Photoswitchable adhesives of spiropyran polymers. *Chem. Mater.* **2022**, *34* ,8289–8296. DOI: 10.1021/acs.chemmater.2c01809.

17) Babra, T. S.; Warriner, C.; Bazin, N.; Hayes, W.; Greenland, B. W. A fluoride degradable crosslinker for debond-on-demand polyurethane based crosslinked adhesives. *Mater. Today Commun.* **2020**, 26, 101777. DOI: 10.1016/j.mtcomm.2020.101777.
